# Divergent kleisin subunits of cohesin specify mechanisms to tether and release meiotic chromosomes

**DOI:** 10.7554/eLife.03467

**Published:** 2014-08-29

**Authors:** Aaron F Severson, Barbara J Meyer

**Affiliations:** 1 Department of Molecular and Cell Biology, Howard Hughes Medical Institute, University of California, Berkeley, Berkeley, United States; 2 Center for Gene Regulation in Health and Disease and Department of Biological, Geological, and Environmental Sciences, Cleveland State University, Cleveland, United States; Institute of Human Genetics, CNRS UPR 1142, France

**Keywords:** cohesin, sister chromatid cohesion, meiosis, gametogenesis, kleisin, aneuploidy, C. elegans

## Abstract

We show that multiple, functionally specialized cohesin complexes mediate the establishment and two-step release of sister chromatid cohesion that underlies the production of haploid gametes. In *C. elegans,* the kleisin subunits REC-8 and COH-3/4 differ between meiotic cohesins and endow them with distinctive properties that specify how cohesins load onto chromosomes and then trigger and release cohesion. Unlike REC-8 cohesin, COH-3/4 cohesin becomes cohesive through a replication-independent mechanism initiated by the DNA double-stranded breaks that induce crossover recombination. Thus, break-induced cohesion also tethers replicated meiotic chromosomes. Later, recombination stimulates separase-independent removal of REC-8 and COH-3/4 cohesins from reciprocal chromosomal territories flanking the crossover site. This region-specific removal likely underlies the two-step separation of homologs and sisters. Unexpectedly, COH-3/4 performs cohesion-independent functions in synaptonemal complex assembly. This new model for cohesin function diverges from that established in yeast but likely applies directly to plants and mammals, which utilize similar meiotic kleisins.

**DOI:** http://dx.doi.org/10.7554/eLife.03467.001

## eLife digest

Most plant and animal cells have a pair of each chromosome: one copy is inherited from the father, the other from the mother. When a cell divides, each daughter cell must receive a copy of all of the original cell's genetic information. To this end, the chromosomes are replicated to form so-called ‘sister chromatids’, which are then segregated equally between the two daughter cells.

In contrast, sex cells such as eggs and sperm (also called gametes) have a single copy of each chromosome. When an egg and a sperm fuse to form a single cell (called a zygote), the zygote ends up with a full set of chromosomes. Gametes are formed by two successive rounds of cell division that occur after the chromosomes are replicated. The first round separates the pairs of chromosomes, and the second separates the sister chromatids to produce the gametes, each of which has half the original amount of genetic information.

If something goes awry in the production of gametes, a zygote can end up with the wrong number of chromosomes. Almost one-third of human zygotes inherit an aberrant complement of chromosomes, and many of these zygotes either fail to survive or develop into offspring with birth defects and developmental disorders.

To ensure that gametes receive the correct number of chromosomes, the sister chromatids remain bound together by a ring-shaped protein complex during the first cell division. Previous studies on how this protein complex—called cohesin—tethers the sister chromatids together were conducted on yeast and mammalian cells. Now, Severson and Meyer show that, in a microscopic worm called *Caenorhabditis elegans*, cohesin functions differently from how it functions in the simpler yeast cells.

Severson and Meyer found that rather than using a single cohesin complex like in yeast, the worms use multiple cohesin complexes that have different versions of one key protein subunit. Changing this single subunit has a major impact on cohesin's function. Consequently, each complex plays a specific role in tethering and then releasing sister chromatids. One of the cohesin complexes is triggered to tether the sister chromatids when the chromosomes replicate. Unexpectedly, another complex only tethers the sisters once breaks occur in the DNA. These breaks allow sister chromatids that are produced from maternally- and paternally-derived chromosomes to cross over and swap genetic material—which increases the genetic diversity of any future offspring. After these genetic swaps occur, the cohesin complexes are then selectively removed by different mechanisms, first to release the pairs of chromosomes and then the sister chromatids.

The findings of Severson and Meyer establish a new model for the mechanisms of chromosome segregation during gamete production. Further studies are now needed to determine the roles and regulation of these protein complexes in other species—including plants and mammals, which use similar cohesin complexes.

**DOI:** http://dx.doi.org/10.7554/eLife.03467.002

## Introduction

In all organisms, faithful segregation of chromosomes during cell division is essential for genome stability. Accurate chromosome transmission is required both for the proliferative cell divisions that occur during mitosis and the sequential divisions that occur during meiosis to reduce genome copy number from two in diploid germline stem cells to one in haploid gametes. Approximately 30% of human zygotes have abnormal chromosomal content at conception due to defects in meiosis. Such aneuploidy is a leading cause of miscarriages and birth defects (Hassold and Hunt, 2001), and is thought to result, in part, from defects in sister chromatid cohesion (SCC) (Chiang et al., 2012; Jessberger, 2012; Nagaoka et al., 2012). SCC tethers replicated sister chromatids during mitosis and meiosis and is critical for accurate chromosome segregation.

SCC is mediated by an evolutionarily conserved protein complex called cohesin. The cohesin complex is composed of two long coiled-coil proteins of the Structural Maintenance of Chromosomes (SMC) family, called Smc1 and Smc3, a non-SMC protein called Scc3, and a fourth subunit called the α-kleisin (Nasmyth and Haering, 2009). Smc1, Smc3 and the kleisin form a tripartite ring proposed to mediate SCC by encircling sister chromatids. The kleisin subunit differs between mitotic and meiotic cohesin complexes. During yeast meiosis, the mitotic kleisin Scc1 is replaced by the meiosis-specific kleisin Rec8 (Klein et al., 1999). This substitution is crucial for the reduction of ploidy.

We recently showed that the dual kleisin model derived for yeast is insufficient to explain how cohesin complexes facilitate the reduction of genome copy number in all organisms, since Rec8 is not the sole meiotic kleisin in many organisms (Severson et al., 2009). Here, we establish a new model: multiple, functionally specialized cohesin complexes that differ in their kleisin subunit perform distinct roles in reducing ploidy. The kleisin influences nearly all aspects of meiotic cohesin function, including how a cohesin complex loads onto meiotic chromosomes, how a complex becomes cohesive once loaded, and when, where and how a complex is removed from chromosomes in meiotic prophase. We first summarize the known roles of meiotic kleisins to provide context for these findings.

Analysis of *rec8* mutants in numerous sexually reproducing organisms showed that Rec8 cohesin is essential for the three key events that are unique to meiosis and underlie the production of haploid gametes (DeVeaux and Smith, 1994; Bhatt et al., 1999; Klein et al., 1999; Watanabe and Nurse, 1999; Buonomo et al., 2000; Pasierbek et al., 2001; Yokobayashi et al., 2003; Bannister et al., 2004; Parra et al., 2004; Chelysheva et al., 2005; Severson et al., 2009; Tachibana-Konwalski et al., 2010; Shao et al., 2011). First, homologous chromosomes become covalently linked through reciprocal exchange of DNA during the process of crossover (CO) recombination. COs promote accurate homolog segregation during anaphase of meiosis I, and Rec8 cohesin is required for efficient CO formation and maintenance. Second, sister chromatids attach to microtubules from the same spindle pole (co-orient) in meiosis I to ensure that spindle forces pull homologs apart but not sister chromatids. Sister chromatids then attach to microtubules from opposite spindle poles (bi-orient) in meiosis II, as they do in mitosis. Rec8 cohesin facilitates co-orientation. Third, spatially-regulated release of meiotic SCC must occur in two steps to allow the sequential separation of homologs in anaphase I and then sisters in anaphase II. Rec8 cohesin is essential for the linkages that tether sisters until anaphase II.

The widely conserved meiotic defects of *rec8* mutants reinforced the view of Rec8 as the sole meiotic kleisin. Our recent work challenged this prevalent view by demonstrating that *Caenorhabditis elegans* gametogenesis requires two nearly identical and functionally redundant predicted α-kleisins, called COH-3 and COH-4 (hereafter, COH-3/4), in addition to REC-8 (Severson et al., 2009). REC-8 and COH-3/4 together mediate meiotic SCC, and severe disruption of SCC occurs only when all three kleisins are removed, suggesting the formation of cohesin complexes that differ in their kleisin subunit. Moreover, REC-8 and COH-3/4 are required for CO recombination. CO recombination fails in *rec-8* single mutants and in *coh-4 coh-3* double mutants, causing homologs to remain detached.

Although REC-8 and COH-3/4 are both required for CO formation and act in concert to mediate SCC, they perform distinct roles in meiotic chromosome segregation (Severson et al., 2009). Unlike REC-8, COH-3/4 cannot co-orient sisters or mediate SCC that persists until anaphase II. Consequently, in *rec-8* mutants, sister chromatids are tethered by COH-3/4-dependent SCC until anaphase I, when they segregate prematurely toward opposite spindle poles (equational division). In contrast, in *coh-4 coh-3* mutants, REC-8 cohesin co-orients sisters during meiosis I and tethers sisters until anaphase II. Consequently, sister chromatids remain together while homologs segregate randomly during anaphase I.

Subsequent to the discovery of COH-3/4 in *C. elegans*, meiotic kleisins similar to COH-3/4 were identified in plants and mammals (Herran et al., 2011; Ishiguro et al., 2011; Lee and Hirano, 2011; Llano et al., 2012; Yuan et al., 2012). The involvement of these kleisins in meiotic SCC likely explains why cohesion persists in *rec8* mutants of *Arabidopsis*, maize, and mouse, as it does in *C. elegans* (Bhatt et al., 1999; Bannister et al., 2004; Chelysheva et al., 2005; Xu et al., 2005; Golubovskaya et al., 2006; Severson et al., 2009). The involvement of multiple kleisins in gametogenesis is therefore widely conserved, and our current study dissects the mechanisms by which the kleisin subunit influences cohesin function.

Here, we show that REC-8 and COH-3/4 are bona fide kleisin subunits of meiotic cohesin complexes, and that the mechanisms that regulate cohesin loading, sister chromatid cohesion, and cohesin removal are strongly affected by the kleisin subunit. We identify factors required for association of REC-8 cohesin, but not COH-3/4 cohesin, with meiotic chromosomes, providing strong evidence of complex-specific loading mechanisms. We show that COH-3/4 cohesin is triggered to become cohesive, and thereby establish SCC, independently of DNA replication and requires the programmed, SPO-11-dependent double-strand DNA breaks (DSBs) that initiate meiotic recombination. This result was not expected, because prior work showed that yeast mitotic cohesin loads onto chromosomes during telophase or G1 of the cell cycle and becomes cohesive only during S phase (Uhlmann and Nasmyth, 1998; Nasmyth and Haering, 2009; Wood et al., 2010). The sole example of replication-independent SCC establishment occurs in mitotically proliferating yeast that suffer DNA damage in G2 or M of the cell cycle (Ström et al., 2007; Unal et al., 2007). The SCC formed in response to DSBs is thought to reinforce the cohesion generated during S phase. Since Rec8 cannot generate SCC in response to DNA damage, damage-induced SCC was thought to occur only in proliferating cells (Heidinger-Pauli et al., 2008). Our data indicate that damage-induced SCC is an essential feature of meiosis. Finally, we show that prior to homolog separation in anaphase I, REC-8 and COH-3/4 cohesins become selectively removed from complementary domains that flank the single CO of each worm chromosome in a separase-independent manner, consistent with their distinct roles in meiotic chromosome segregation: COH-3/4 becomes enriched where SCC is released at anaphase I and REC-8 becomes enriched where sister chromatids co-orient and SCC persists until anaphase II. Because REC-8 alone can co-orient sisters and mediate SCC that persists after anaphase I, this reciprocal pattern of cohesin removal may facilitate or underlie the stepwise separation of homologs and sister chromatids. This finding contrasts with the two-step cohesion release mechanism of yeast that utilizes only Rec8 and factors like Mei-S332/Shogushin to protect centromeric Rec8 cohesin from degradation during anaphase I, thereby ensuring sister cohesion until anaphase II. Our findings not only reveal unanticipated features of meiosis in *C. elegans*, but also establish models of meiotic cohesin function applicable to gametogenesis in plants and mammals.

## Results

### COH-3 and COH-4 are bona fide subunits of meiotic cohesin complexes

**Figure 1.**
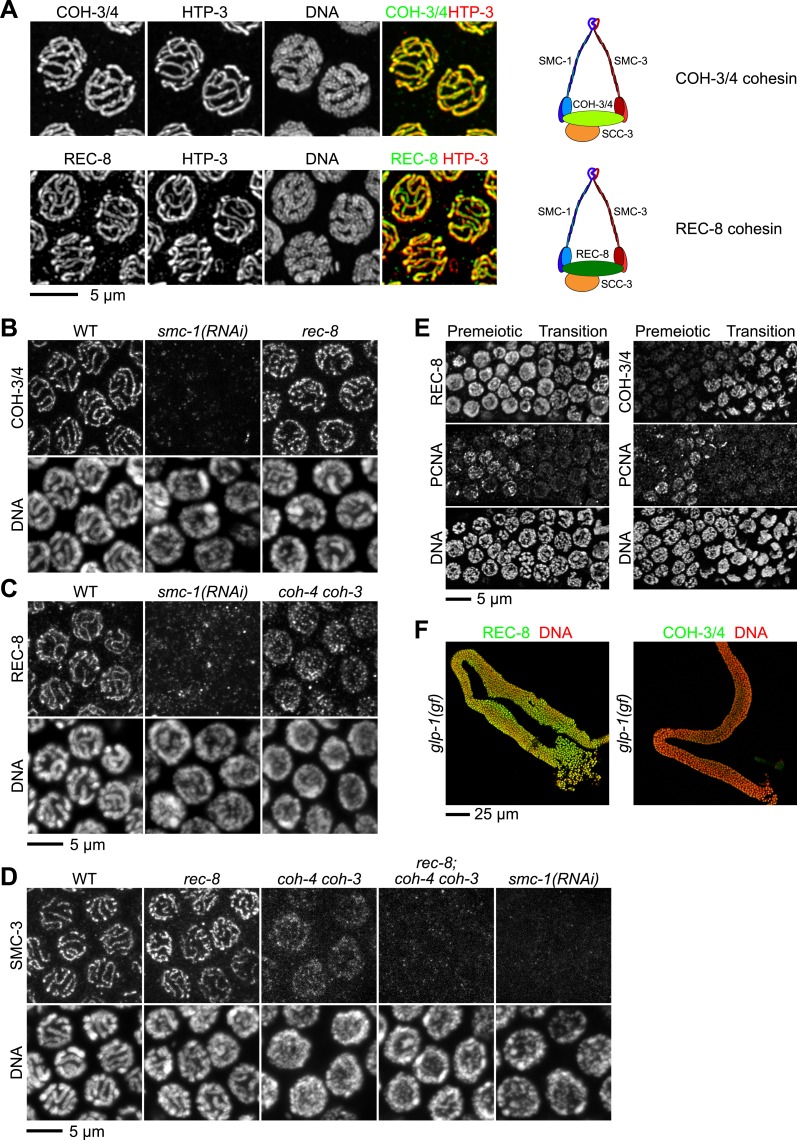
Multiple cohesin complexes that differ in their kleisin subunit bind to *C. elegans* meiotic chromosomes. Interdependent loading of REC-8 and COH-3/4 with cohesin SMC proteins is demonstrated. Shown are Z-projected confocal sections through pachytene nuclei (**A**–**D**), the distal region of the gonad (**E**), and entire dissected gonads (**F**). (**A**) The predicted α-kleisins REC-8 and COH-3/4 are present along synapsed homologs in pachytene nuclei of wild-type animals and co-localize with the axis protein HTP-3, as expected for meiotic kleisins. COH-3/4 (**B**) and REC-8 (**C**) both require SMC-1 for their association with meiotic chromosomes but bind chromosomes independently. (**D**) SMC-3 associates with chromosomes of *rec-8* and *coh-4 coh-3* mutants, but SMC-3 staining is undetectable in kleisin triple mutants and *smc-1(RNAi)* animals. (**E**) The distal region of the gonad holds nuclei undergoing mitotic proliferation and premeiotic DNA replication (Premeiotic Zone) and nuclei that have entered prophase of meiosis I (Transition Zone). REC-8 is strongly expressed in all germline nuclei, including S phase nuclei, which express GFP::PCN-1. In contrast, COH-3/4 staining is undetectable in GFP::PCN-1 positive nuclei and first appears on meiotic chromosomes in the transition zone, indicating that COH-3/4 cohesin becomes cohesive independently of DNA replication. (**F**) *glp-1(gf)* mutations prevent initiation of meiosis; consequently, the gonad fills with mitotically proliferating germ cell nuclei. Robust expression of REC-8, but not COH-3/4, is detected in the mitotic nuclei of *glp-1(gf)* worms, indicating that COH-3/4 is first expressed during meiosis. **DOI:** http://dx.doi.org/10.7554/eLife.03467.003

During *C. elegans* meiosis, the α-kleisin paralogs REC-8 and COH-3/4 function in sister chromatid cohesion (SCC) but perform specialized functions (Severson et al., 2009), suggesting their participation in independent cohesin complexes (Severson et al., 2009). To test this hypothesis, we generated antibodies that recognize both COH-3 and COH-4 and assessed whether COH-3/4 associate with meiotic chromosomal axes, as expected for cohesin subunits. Indeed, REC-8 and COH-3/4 co-localized with the chromosomal axis protein HTP-3 (Goodyer et al., 2008) in pachytene nuclei of wild-type animals (Figure 1A). The COH-3/4 antibody recognizes COH-3 and COH-4 specifically, since staining was undetectable in *coh-4 coh-3* double mutants, but strong staining persisted in both single mutants (Figure 1—figure supplement 1A), as expected from the complete genetic redundancy of the *coh-4* and *coh-3* genes (Severson et al., 2009).

**Figure 1—figure supplement 1.**
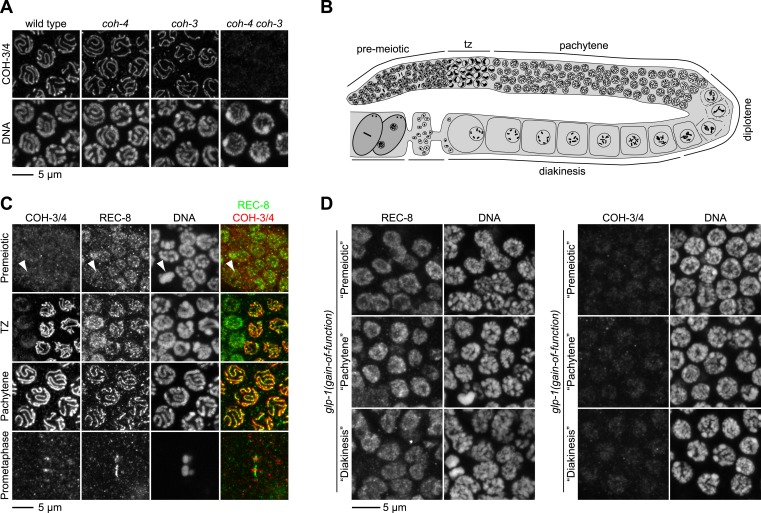
REC-8 accumulates in hermaphrodite gonads prior to the initiation of meiosis, while COH-3/4 becomes detectable only in meiotic nuclei. (**A**) Rabbit polyclonal antibodies recognize both COH-3 and COH-4. COH-3/4 antibodies label meiotic chromosomes in wild-type animals as well as *coh-3* and *coh-4* single mutants. Antibody staining is undetectable only in *coh-4 coh-3* double mutants. (**B**) Cartoon showing that premeiotic nuclei and nuclei in various stages of meiosis occupy distinct, predictable regions of the gonad in wild-type animals. (**C**) Confocal micrographs of germline nuclei stained with DAPI and antibodies to REC-8 and COH-3/4. COH-3/4 are undetectable in the distal-most region of the gonad, which contains mitotically-cycling germline stem cells and nuclei in premeiotic S phase. Arrowheads indicate a metaphase figure. COH-3/4 appear abruptly on chromosomes at the onset of meiotic prophase (transition zone, TZ), and COH-3/4 are detected along the entire chromosomal axis in pachytene. By prometaphase of meiosis I, COH-3/4 are detected only at the short arm. The pattern of COH-3/4 localization differs from that of REC-8 in two important ways. First, REC-8 is expressed in premeiotic nuclei, although it is unclear whether REC-8 is bound to chromosomes at this stage. Second, while COH-3/4 become restricted to the short arm by prometaphase I, REC-8 is removed from the short arm but persists at the long arm. (**D**) Gain-of-function alleles of *glp-1* prevent initiation of meiosis, and consequently, the germline fills with mitotically proliferating nuclei. High magnification confocal images of regions of the gonad that would contain premeiotic, pachytene, or diakinesis nuclei in wild-type animals are shown. REC-8 is highly expressed in all of these nuclei, but COH-3/4 are not. **DOI:** http://dx.doi.org/10.7554/eLife.03467.004

As subunits of distinct cohesin complexes, COH-3/4 and REC-8 are expected to bind chromosomes independently of each other and to require the SMC subunits for their chromosomal association. Moreover, SMC staining should persist in *rec-8* single and *coh-4 coh-3* double mutants but not in *rec-8; coh-4 coh-3* triple mutants (hereafter called kleisin triple mutants). These expectations were met (Figure 1B–D). Long tracks of COH-3/4 (Figure 1B) and SMC-3 (Figure 1D; Chan et al., 2003) staining were evident on meiotic chromosomes of *rec-8* single mutants, and both REC-8 (Figure 1C) and SMC-3 (Figure 1D) persisted on chromosomes of *coh-4 coh-3* mutants. However, levels of REC-8 and SMC-3 were reduced in *coh-4 coh-3* double mutants compared to wild-type animals, and both proteins appeared in dispersed puncta rather than in linear structures, as previously noted for the axial element HTP-3 (Severson et al., 2009). The disorganized localization and reduced staining intensity of REC-8 and SMC-3 reflect the failure to form continuous chromosomal axes. Loss of COH-3/4 binding also reduces SMC-3 levels. In contrast, SMC-3 was nearly undetectable on meiotic chromosomes of kleisin triple mutants (Figure 1D). In converse experiments, binding of REC-8 (Figure 1C) and COH-3/4 (Figure 1B) to meiotic chromosomes was severely disrupted in *smc-1(RNAi)* animals, as was binding of SMC-3 (Figure 1D). These results provide strong evidence that REC-8 and COH-3/4 associate with chromosomes as subunits of independent meiotic cohesin complexes that differ in their kleisin subunit (Figure 1A).

### COH-3 and COH-4 accumulate on chromosomal axes after premeiotic DNA replication

In proliferating cells, cohesin loading and SCC establishment are temporally separate events: Scc1 cohesin loads onto chromosomes prior to S phase and becomes cohesive during DNA replication (Guacci et al., 1997; Michaelis et al., 1997; Losada et al., 1998; Uhlmann and Nasmyth, 1998). *C. elegans* REC-8 cohesin appears to behave similarly, since REC-8 accumulates before premeiotic S phase (Pasierbek et al., 2001; Hayashi et al., 2007; Severson et al., 2009). To our surprise, COH-3/4 cohesin behaves differently: COH-3/4 become detectable during meiotic prophase, after completion of premeiotic replication.

To determine the precise timing of REC-8 and COH-3/4 accumulation, we examined the staining of each kleisin during the different stages of germ-cell development. In *C. elegans*, changes in chromosomal morphology and nuclear position distinguish germ-cell nuclei undergoing mitotic proliferation or premeiotic DNA replication from nuclei in meiotic prophase I (Figure 1—figure supplement 1B). Chromosomes are dispersed in premeiotic nuclei, which occupy the most distal region of the gonad. Upon initiation of meiosis, chromosomes cluster in a crescent on one side of the nucleus, opposite the nucleolus, in a region of the gonad called the transition zone (Francis et al., 1995; MacQueen and Villeneuve, 2001). Nuclei in this region are in the leptotene and zygotene stages of prophase I, when synaptonemal complexes (SCs) assemble between homologs. In leptotene, linear structures called axial elements (AEs) form along the length of meiotic chromosomes. In zygotene, central region (CR) proteins assemble between homologous AEs, tethering homologs along their lengths in a process called synapsis. Chromosomes remain clustered until pachytene, when the fully-synapsed homologs redistribute around the nuclear periphery.

As shown previously, we detected REC-8 in all germ-cell nuclei of the gonad, including premeiotic nuclei, consistent with SCC establishment in premeiotic S phase (Figure 1E, Figure 1—figure supplement 1C) (Pasierbek et al., 2001; Hayashi et al., 2007; Severson et al., 2009). In contrast, COH-3/4 was not detected in premeiotic nuclei, but intense COH-3/4 staining appeared abruptly on meiotic chromosomal axes in the transition zone and persisted through prophase I, suggesting that COH-3/4 first accumulates at the onset of meiosis (Figure 1E, Figure 1—figure supplement 1C). Two additional lines of evidence supported this conclusion. First, COH-3/4 staining was not detected in nuclei that expressed PCNA, an S phase-specific DNA polymerase processivity factor used for mitotic and premeiotic DNA replication (Figure 1E). Second, in animals carrying a *glp-1* gain-of-function allele that blocks the initiation of meiosis (Berry et al., 1997), REC-8, but not COH-3/4, was strongly expressed in the mitotically proliferating nuclei that filled the entire gonad (Figure 1F, Figure 1—figure supplement 1D). Thus, COH-3/4 associates with chromosomes after premeiotic S phase is complete, suggesting that COH-3/4 and REC-8 cohesins may load onto chromosomes and become cohesive by different mechanisms.

### The kleisin subunit determines the mechanism of cohesin loading

**Figure 2.**
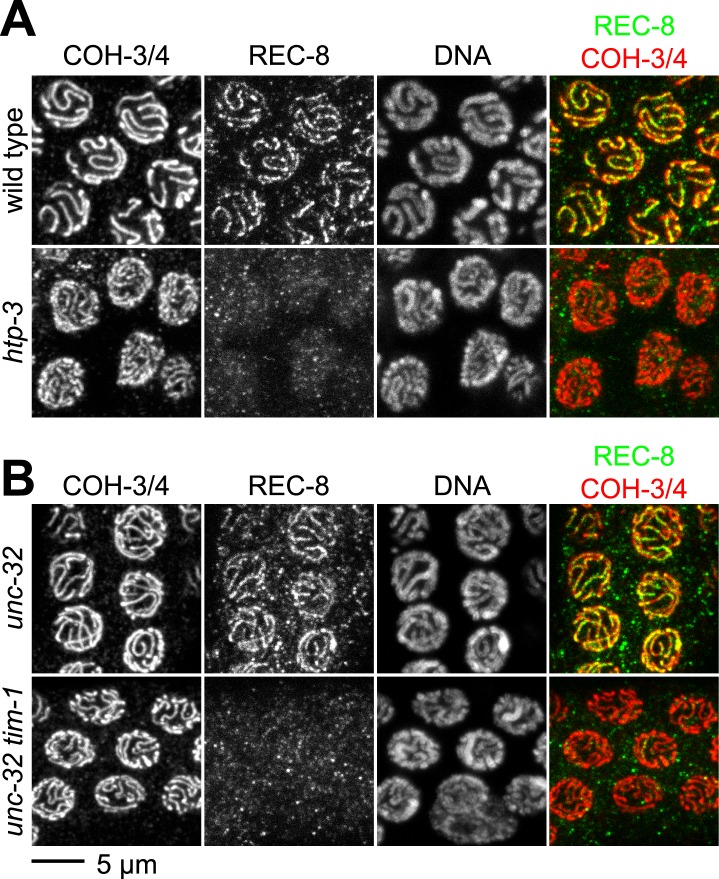
The kleisin subunit determines mechanisms of cohesin loading. Confocal micrographs of pachytene nuclei reveal that the axial element protein HTP-3 (**A**) and the Timeless ortholog TIM-1 (**B**) are both essential for REC-8 cohesin loading, but neither protein is needed for COH-3/4 cohesin loading. **DOI:** http://dx.doi.org/10.7554/eLife.03467.005

The cohesin loading factors identified to date, exemplified by the heterodimeric Scc2/Scc4 complex, are required for the loading of all cohesin complexes examined, regardless of subunit composition (Ciosk et al., 2000; Gillespie and Hirano, 2004; Takahashi et al., 2004; Lightfoot et al., 2011). Our finding that REC-8 accumulates in both premeiotic and meiotic nuclei, but COH-3/4 accumulates only in meiotic nuclei, suggested that REC-8 and COH-3/4 cohesins might load by different mechanisms. We therefore examined COH-3/4 loading in two mutant strains in which REC-8 was undetectable on meiotic chromosomes but SCC persisted: *htp-3* mutants lacking the HORMA domain AE protein HTP-3 (Goodyer et al., 2008; Severson et al., 2009), and *tim-1* mutants lacking the *C. elegans* TIMELESS homolog TIM-1 (Chan et al., 2003) (Figure 2). TIMELESS was initially identified in *Drosophila* as a factor involved in circadian rhythms, but TIMELESS orthologs were subsequently shown to function during DNA replication (Gotter et al., 2007). In both mutants, REC-8 was detected at normal levels in premeiotic nuclei but failed to associate with meiotic chromosomes (Chan et al., 2003; Severson et al., 2009). In contrast, COH-3/4 loading appeared normal in *htp-3* and *tim-1* mutants (Figure 2). Thus, specific factors likely promote the differential loading of cohesin complexes, and the kleisin subunit specifies the mechanism by which a complex initially associates with meiotic chromosomes.

### An assay to quantify SCC defects

**Figure 3.**
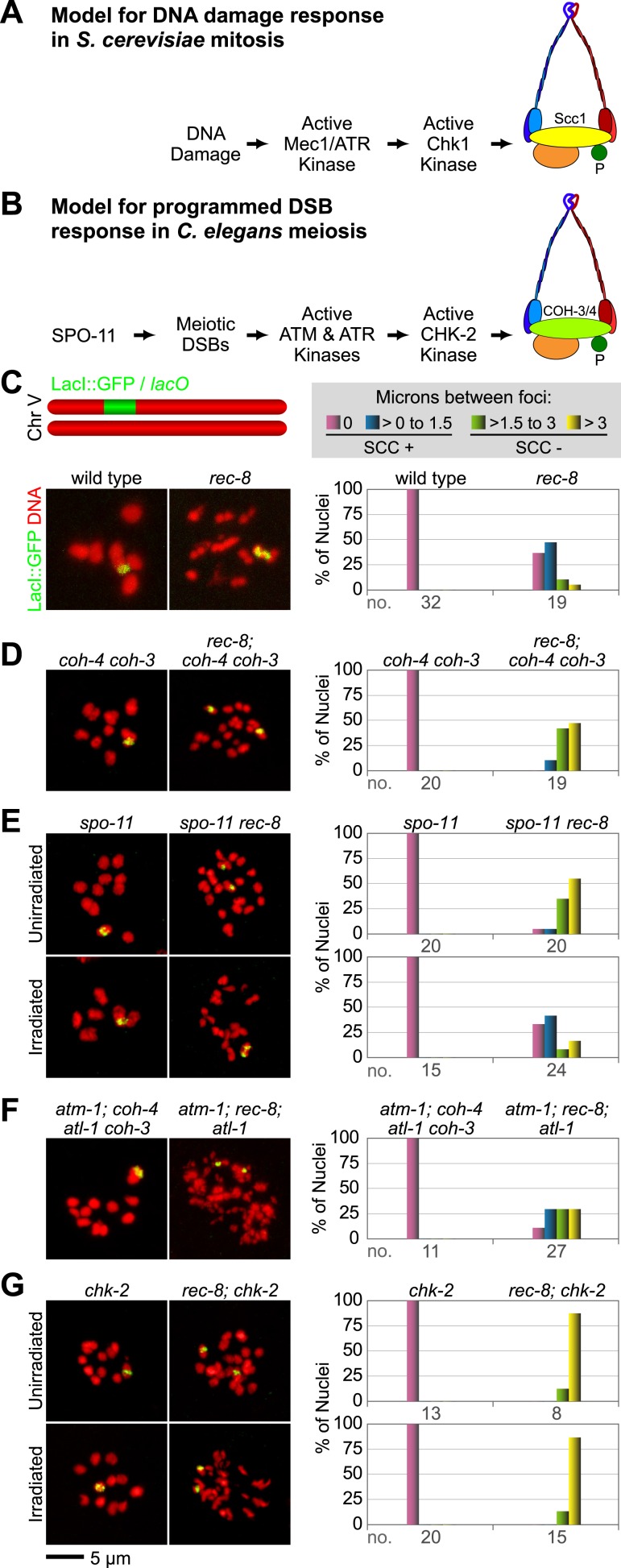
A conserved mechanism initiates SCC in response to programmed DSBs in *C. elegans* meiosis and exogenous DNA breaks in budding yeast mitosis. (**A**) In *S. cerevisiae*, DNA damage in G2/M activates ATR and Chk1, resulting in Scc1 phosphorylation and SCC establishment. (**B**) A model for SCC establishment by COH-3/4 cohesin. DSBs created by SPO-11 activate ATM/ATR and CHK-2, leading to COH-3/4 phosphorylation and generation of SCC. (**C**–**G**) Data supporting the model in (**B**). Images on the left show projected Z-sections through entire diakinesis nuclei stained with LacI::GFP (green) and DAPI (red). LacI::GFP bound to a heterozygous *lacO* array integrated into chromosome V reveals whether sisters are held together by SCC. Charts on the right show quantification of distances between LacI::GFP foci. 0 µm indicates that discrete GFP foci could not be resolved. no. = number of nuclei scored. (**C**) LacI::GFP labels a single bivalent in wild-type animals, and the two sisters of a single univalent in *rec-8* mutants. (**D**) Sister chromatids are held together by REC-8-dependent SCC in *coh-4 coh-3* double mutants, but are apart in kleisin triple mutants. (**E**) Sister chromatids are held together by SCC in *spo-11* mutants, but not in *spo-11 rec-8* mutants. DSBs induced by γ-irradiation restore SCC in *spo-11 rec-8* mutants. (**F**) Sisters are apart and extensive chromosomal fragmentation and rearrangement occurs in *atm-1; rec-8; atl-1* animals, but not in *atm-1; coh-4 atl-1 coh-3* mutants. (**G**) Cohesion between sisters is not established in *rec-8; chk-2* mutants. Irradiation of *rec-8; chk-2* mutants does not restore SCC but does induce chromosome fragmentation and rearrangement, demonstrating a role for CHK-2 in SCC establishment that is downstream of DSB formation. CHK-1 is not required for COH-3/4-dependent SCC (Figure 3—figure supplement 3B). **DOI:** http://dx.doi.org/10.7554/eLife.03467.006

To determine whether REC-8 and COH-3/4 cohesins also differ in their requirements for triggering SCC, we first developed a reliable assay for evaluating SCC. We assessed sister-chromatid tethering in strains carrying an array of *lac* operator repeats (*lacO)* that had been integrated into one of the two chromosome V homologs (Figure 3C,D) (Gonzalez-Serricchio and Sternberg, 2006). LacI::GFP binding to *lacO* repeats uniquely marks the two sister chromatids of the homolog harboring the array (Belmont and Straight, 1998; Gonzalez-Serricchio and Sternberg, 2006). The ability to identify a specific pair of sister chromatids and measure the distance between them permits more accurate quantification of SCC defects than previous methods reliant on fluorescence in situ hybridization or counting of DAPI-staining bodies.

**Figure 3—figure supplement 1.**
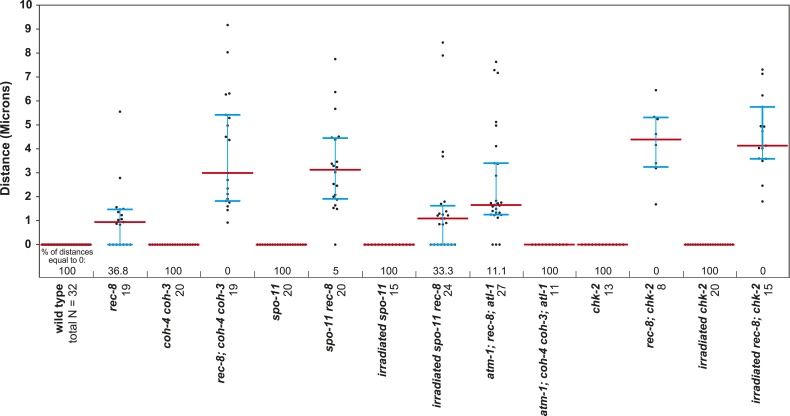
Beeswarm plots show individual distances between LacI::GFP foci in diakinesis nuclei. Red horizontal lines indicate the median, and blue horizontal lines indicate the interquartile range. A distance of >1.5 microns indicates defective SCC. **DOI:** http://dx.doi.org/10.7554/eLife.03467.007

**Figure 3—figure supplement 2.**
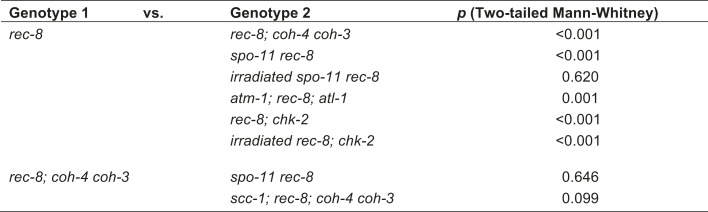
Table of significance values for distances between LacI::GFP foci in diakinesis nuclei. **DOI:** http://dx.doi.org/10.7554/eLife.03467.008

**Figure 3—figure supplement 3.**
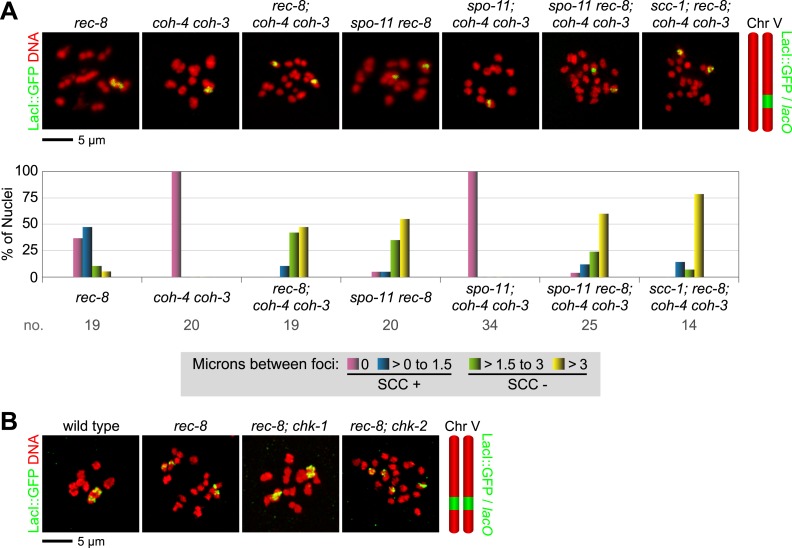
REC-8 and COH-3/4 cohesin tether SCC by different mechanisms. Shown are projected confocal images of entire diakinesis nuclei stained with DAPI (red) and LacI::GFP (green). SCC was assessed by the distribution of LacI::GFP bound to a *lacO* array integrated into one (**A**) or both (**B**) chromosome V homologs. Bar charts show quantification of distances between LacI::GFP foci. 0 µm indicates that discrete GFP foci could not be resolved. no. = number of nuclei scored. (**A**) SPO-11-dependent DSBs trigger SCC mediated by COH-3/4 but not REC-8 cohesin. In *rec-8* single mutants, the sister chromatids of each homolog are tethered together by COH-3/4-dependent SCC. Similarly, sisters are held together by REC-8-dependent SCC in *coh-4 coh-3* double mutants. Sisters are detached in diakinesis nuclei of *spo-11 rec-8* double mutants, *rec-8; coh-4 coh-3* triple mutants, and *spo-11 rec-8; coh-4 coh-3* quadruple mutant animals, and the frequency of detachment and the distance between sisters are similar in all three genotypes. In contrast, disrupting SPO-11 function in *coh-4 coh-3* double mutants does not lead to cohesion defects. (**B**) COH-3/4-dependent SCC requires CHK-2, but not CHK-1. Diakinesis nuclei of *rec-8; chk-1(RNAi)* animals resemble those of *rec-8* single mutants, while sisters are detached in diakinesis nuclei of *rec-8; chk-2(RNAi)* worms. Thus, CHK-2, but not CHK-1, is required for the establishment of COH-3/4 dependent SCC. **DOI:** http://dx.doi.org/10.7554/eLife.03467.009

We validated the assay and established a quantitative metric for SCC by measuring the distance between sister chromatids in nuclei of (1) wild-type animals in which both REC-8 and COH-3/4 cohesins tether sisters, (2) kleisin triple mutants in which SCC is not established (Severson et al., 2009), and (3) *rec-8* single or *coh-4 coh-3* double mutants in which SCC is achieved by a single type of cohesin. Because REC-8 and COH-3/4 cohesins are both sufficient to tether sisters in diakinesis nuclei (Severson et al., 2009), the contribution of REC-8 to SCC could only be assessed in *coh-4 coh-3* mutants, and the contribution of COH-3/4 could only be assessed in *rec-8* mutants. Our initial analysis focused on nuclei in late diakinesis, the final stage of meiotic prophase I, because diakinesis chromosomes are highly compacted and the nuclear volume is much greater than that in earlier stages of meiosis.

As expected, a single GFP focus was detected in 100% of diakinesis nuclei of wild-type worms and *coh-4 coh-3* double mutants (Figure 3C,D and Figure 3—figure supplement 1), consistent with the finding that REC-8 cohesin is sufficient to tether and co-orient sister chromatids (Severson et al., 2009). LacI::GFP also labeled a single detached homolog (univalent) in most diakinesis nuclei of *rec-8* worms; however, two discrete GFP foci could usually be detected within the univalent (Figure 3C and Figure 3—figure supplement 1). This finding was also anticipated because sister chromatids bi-orient in *rec-8* mutants, and the univalents adopt a dumbbell shape in which the two sister chromatids can usually be resolved (Figure 3C; Severson et al., 2009). In 84% of *rec-8* nuclei, either a single GFP focus was detected or two foci were detected within one univalent at a spacing of ≤1.5 µm (Figure 3C). In contrast, only 10% of diakinesis nuclei in kleisin triple mutants had LacI::GFP foci separated by ≤ 1.5 µm (Figure 3D and Figure 3—figure supplements 1 and 2) (Severson et al., 2009). Based on these measurements, we defined diakinesis nuclei with GFP foci ≤1.5 µm apart as having sisters tethered by meiotic SCC, and nuclei with GFP foci >1.5 µm apart as having sisters separated due to defective SCC. The number and morphology of DAPI-staining bodies in diakinesis nuclei of each genotype examined indicated that other chromosomes behaved similarly to chromosome V (Figure 3C,D). Thus, the distance between LacI::GFP spots is a reliable measure of the global status of SCC in diakinesis nuclei.

### SPO-11-dependent DSBs are essential for COH-3/4-dependent SCC

Using the SCC assay, we assessed whether COH-3/4 cohesin becomes cohesive independently of DNA replication, as suggested by the lack of detectable COH-3/4 in PCNA-positive nuclei (Figure 1E). Because the sole example of replication-independent SCC establishment occurs during yeast G2/M phase in response to DNA breaks (Ström et al., 2007; Unal et al., 2007), we hypothesized that the programmed, SPO-11-dependent DSBs used to initiate CO recombination might trigger COH-3/4 to become cohesive.

**Figure 4.**
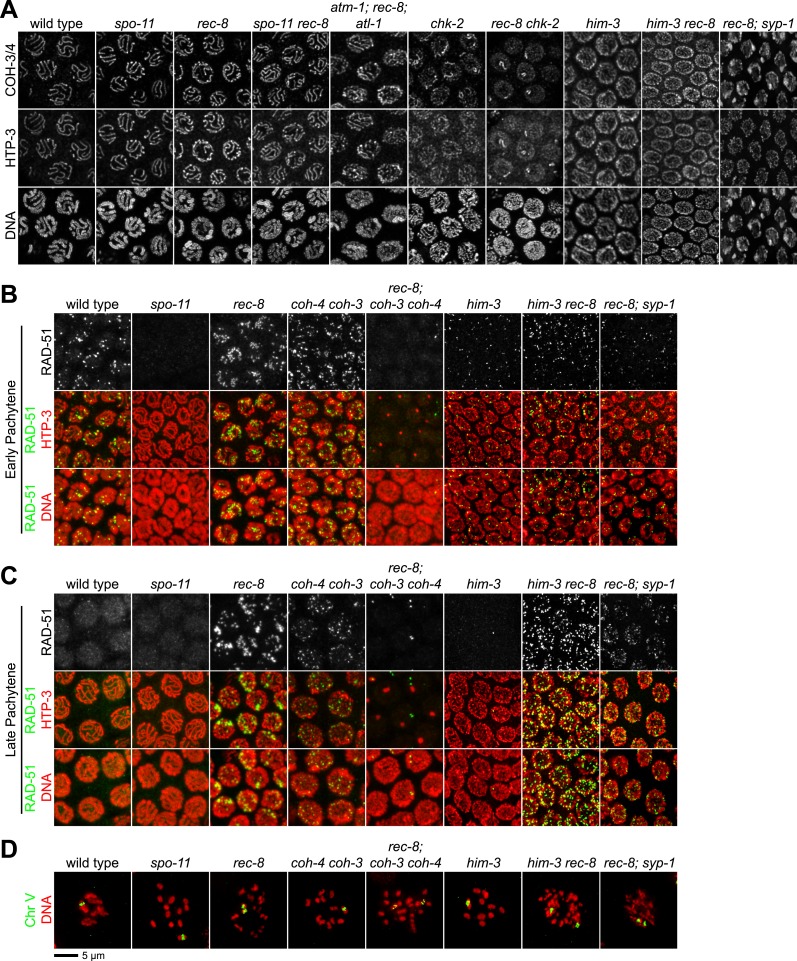
Analysis of COH-3/4 cohesin loading and DSB formation and repair in SCC-defective worms. (**A**) Imaging of pachytene nuclei stained with antibodies to COH-3/4 and HTP-3 demonstrated that COH-3/4 associates with meiotic axes in most mutants that fail to establish COH-3/4-dependent SCC. Although COH-3/4 associates with chromosomes of *him-3 rec-8* animals, the intensity of COH-3/4 signal is less than that detected in *him-3* single mutants, which, in turn, is less than that in wild-type animals (See also Figure 4—figure supplement 2). A reduction in signal is also true of DAPI and HTP-3, which loads onto chromosomes independently of HIM-3 (Goodyer et al., 2008; Severson et al., 2009). Thus, the strong staining of COH-3/4 and HTP-3 observed in wild-type nuclei likely results from the close association of the four chromatid axes via synapsis and SCC, while the reduced staining in *him-3* mutants likely results from homolog separation due to defective synapsis, and in *him-3 rec-8* mutants from homolog and sister separation due to defective synapsis and SCC. Consistent with this model, a similar reduction in the intensity of COH-3/4 and HTP-3 staining was detected in *rec-8* animals also lacking the CR protein SYP-1, which is dispensable for chromosomal loading of all known AE proteins (MacQueen et al., 2002; AFS unpublished data). (**B** and **C**) Confocal images of early (**B**) and late (**C**) pachytene nuclei stained with DAPI (red) and antibodies to DSB marker RAD-51 (green). Abundant RAD-51 foci are detected in *him-3 rec-8* and *rec-8; syp-1* mutants, indicating that DSBs are formed. RAD-51 staining persists abnormally late in these mutants, and chromosomal fragmentation and fusions are evident in diakinesis nuclei (**D**) stained with DAPI (red) and LacI::GFP (green) as in Figure 3C. Thus, establishment of COH-3/4-dependent SCC is essential for homology-directed DSB repair in animals homozygous for a *rec-8* deletion. Remarkably, such rearrangements are not detected in kleisin triple mutants. Explaining this finding, few RAD-51 foci form in kleisin triple mutants. Those that do appear in late pachytene, well after DSBs are repaired in wild-type animals. **DOI:** http://dx.doi.org/10.7554/eLife.03467.010

We found sisters to be apart in 90% of diakinesis nuclei of *spo-11 rec-8* double mutants, indicating that COH-3/4 requires SPO-11 to tether sister chromatids (Figure 3E and Figure 3—figure supplements 1 and 2). In contrast, sisters could not be resolved in any nuclei of *spo-11; coh-4 coh-3* triple mutants, indicating that REC-8 cohesin becomes cohesive independently of SPO-11 (Figure 3—figure supplement 3A). The SCC disruption in *spo-11 rec-8* mutants did not result from a defect in COH-3/4 cohesin loading, since levels of bound COH-3/4 were similar in wild-type animals, *spo-11 rec-8* double mutants, and *spo-11* or *rec-8* single mutants (Figure 4A).

**Figure 4—figure supplement 1.**
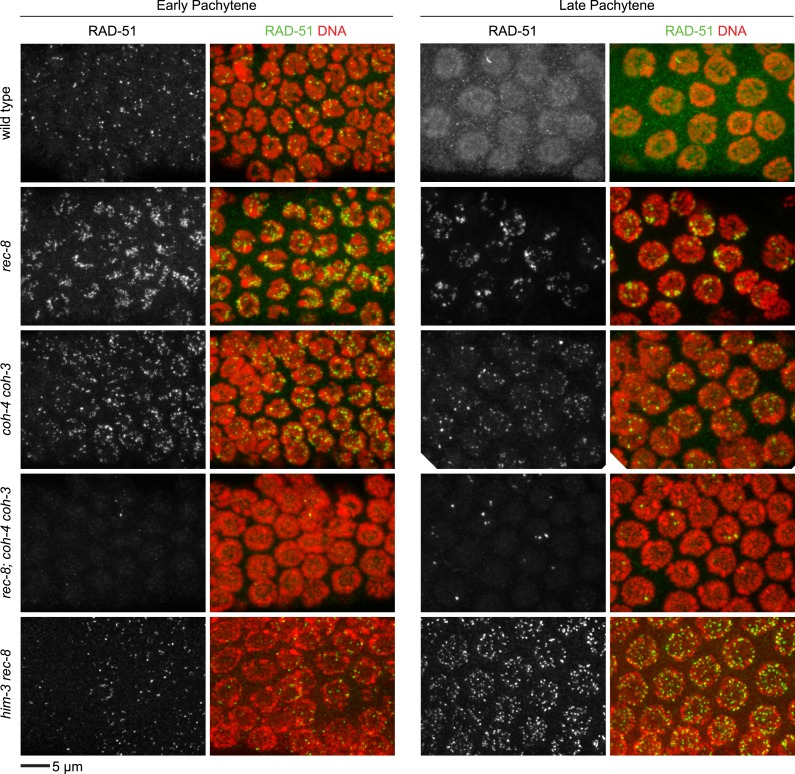
Enlargements of early and late pachytene nuclei of wild-type and mutant animals stained with RAD-51 antibodies. Abundant RAD-51 foci are present in nuclei of all genotypes, except in *rec-8; coh-4 coh-3* mutants, which have a severely reduced number of foci. RAD-51 foci are very rarely present in early pachytene nuclei, and occasional foci appear in late pachytene nuclei. **DOI:** http://dx.doi.org/10.7554/eLife.03467.011

**Figure 4—figure supplement 2.**
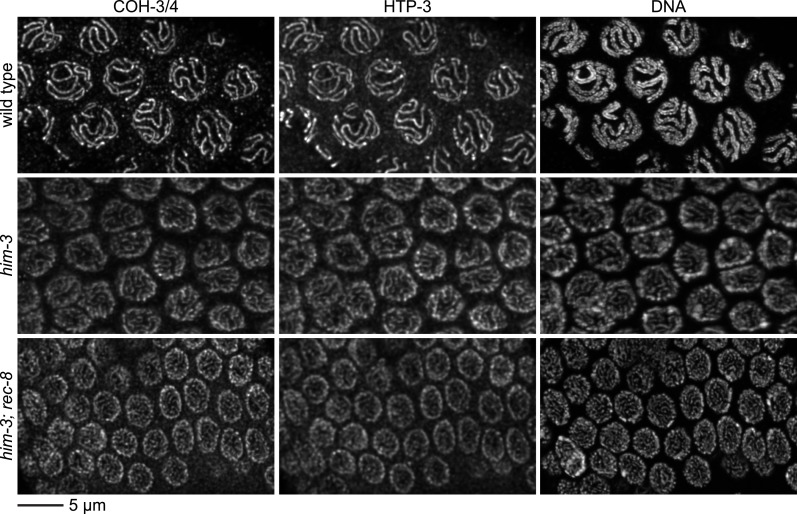
Enlargements of pachytene nuclei from wild-type animals and *him-3* or *him-3; rec-8* mutants stained with DAPI and antibodies to COH-3/4, HTP-3. COH-3/4 and HTP-3 associate with pachytene chromosomes but the levels are reduced in mutants relative to wild-type animals, likely reflecting a failure of homolog synapsis in *him-3* single mutants and defective synapsis and SCC in *him-3 rec-8* mutants. **DOI:** http://dx.doi.org/10.7554/eLife.03467.012

The requirement for SPO-11 in tethering sister chromatids in *rec-8* mutants reflects a need for DSBs, since SCC was restored in *spo-11 rec-8* mutants treated with γ-irradiation. Prior experiments showed that γ-irradiation restored CO recombination in *spo-11* mutants and suppressed lethality (Dernburg et al., 1998). In our experiments, 12 Gy of γ-irradiation triggered CO recombination between most homolog pairs of *spo-11* mutants without causing chromosomal fragmentation or fusion (Figure 3E). That dose also restored SCC: sisters were tethered in 75% of irradiated *spo-11 rec-8* double mutants (Figure 3E and Figure 3—figure supplements 1 and 2). Thus, programmed meiotic DSBs are essential for COH-3/4-dependent SCC, and the kleisin subunit determines whether a cohesin requires DSBs to establish cohesion. Moreover, our data suggest that meiotic SCC is established in successive waves: during premeiotic DNA replication, the nascent sister chromatids are tethered by Watson-Crick base pairing in unreplicated regions and by REC-8 cohesin in replicated regions. Subsequently, DSBs trigger COH-3/4 cohesin to become cohesive during meiosis. This second wave of SCC establishment reinforces the cohesion generated during DNA replication. Evidence supporting this model is described in the following paragraphs.

### A conserved mechanism mediates DSB-induced SCC in yeast mitosis and worm meiosis

The essential role for DSBs in COH-3/4-dependent meiotic SCC suggested that the pathway for establishing SCC in response to DNA damage during yeast G2/M phase might similarly trigger COH-3/4-mediated SCC during nematode meiosis. In the yeast model for DSB-induced SCC (DI-SCC), DNA breaks activate the Mec1/ATR kinase. Mec1 in turn stimulates the Chk1 kinase to phosphorylate the mitotic kleisin Scc1 on serine 83, which triggers cohesin to become cohesive (Figure 3A; Heidinger-Pauli et al., 2008). Yeast Rec8 lacks the Chk kinase phosphorylation site and consequently cannot become cohesive in response to DNA damage. Thus, DI-SCC was thought to occur only during G2/M of the mitotic cell cycle.

To determine whether a similar signaling cascade occurs in *C. elegans* meiosis (Figure 3B), we first asked whether ATM and the related kinase ATR (ATM-1 and ATL-1, respectively) are required for the COH-3/4-dependent SCC that tethers sisters in *rec-8* mutants. Because ATM-1 and ATL-1 perform partially redundant functions in *C. elegans* (Garcia-Muse and Boulton, 2005), we examined cohesion in *atm-1; atl-1* double mutants. Sisters were apart in 59% of diakinesis nuclei in *atm-1; rec-8; atl-1(RNAi)* mutants (Figure 3F and Figure 3—figure supplements 1 and 2). The persistence of cohesion between some chromatids likely results from incomplete ATL-1 depletion. Unfortunately, SCC cannot be examined in *atm-1; rec-8* animals carrying an *atl-1* null allele due to severe defects in gonadal development. Importantly, the SCC defects in *atm-1; rec-8; atl-1(RNAi)* mutants did not result from impaired COH-3/4 cohesin loading (Figure 4A). Furthermore, sisters were always tethered in *coh-4 coh-*3 mutants deficient in ATM-1 and ATL-1 (100%, Figure 3F and Figure 3—figure supplement 1). We conclude that ATM-1 and ATL-1 are required to trigger the cohesiveness of COH-3/4 cohesin, but not REC-8 cohesin.

We next asked whether CHK-1 or the paralogous kinase CHK-2 is required for SCC establishment by COH-3/4 cohesin. Diakinesis nuclei of *rec-8; chk-1(RNAi)* worms had 12 univalents, and LacI::GFP staining showed no sister separation, indicating that CHK-1 kinase is not required (Figure 3—figure supplement 3B). In contrast, sisters were apart in 100% of *rec-8; chk-2* nuclei (Figure 3G, Figure 3—figure supplements 2, 3 and 3B). Because CHK-2 is required for the formation of SPO-11-dependent DSBs (Alpi et al., 2003; Martinez-Perez and Villeneuve, 2005), the failure to establish COH-3/4-dependent SCC in *rec-8; chk-2* animals could have resulted from the absence of DSBs rather than a failure to respond to DSBs. However, exposure of *rec-8; chk-2* mutants to the dose of γ-irradiation that restored SCC in *spo-11 rec-8* worms failed to restore SCC in *rec-8; chk-2* mutants. Sisters remained apart (100%), and chromosome fragments were evident, indicating defective repair of DSBs by homologous recombination (Figure 3G and Figure 3—figure supplements 1 and 2). Thus, DSBs, ATM-1/ATL-1 and CHK-2, but not CHK-1, are required for COH-3/4-dependent SCC. The lower levels of COH-3/4 in *chk-2* single and *rec-8; chk-2* double mutants compared to those in wild-type, *spo-11 rec-8*, and *atm-1; rec-8; atl-1* animals also suggest the possibility that CHK-2 is required for COH-3/4 cohesin loading in addition to triggering SCC.

Our results provide strong evidence that a conserved pathway establishes SCC in response to DSBs in yeast mitosis and in *C. elegans* meiosis. However, the DI-SCC response during yeast G2/M is initiated by stochastic DSBs and is a secondary mechanism of SCC establishment by Scc1 cohesin, while the DI-SCC response during meiosis is triggered by programmed DSBs that initiate CO recombination and is the primary mechanism for SCC establishment by COH-3/4 cohesin.

### A meiotic role for mitotic kleisin SCC-1

Analysis of SCC in diakinesis demonstrated the essential role of REC-8 and COH-3/4 in tethering sister chromatids. We next analysed SCC in pachytene nuclei to assess the role of REC-8 and COH-3/4 in triggering SCC. Our analysis confirmed the central role of these meiotic kleisins in establishing SCC and also revealed the unexpected finding that some sister chromatid linkages can also be formed independently of REC-8 and COH-3/4. Due to the smaller size and different chromosomal structure of pachytene nuclei, distance measurements were divided into 1 µm bins, and sisters were scored as tethered if separated by ≤ 1 µm.

**Figure 5.**
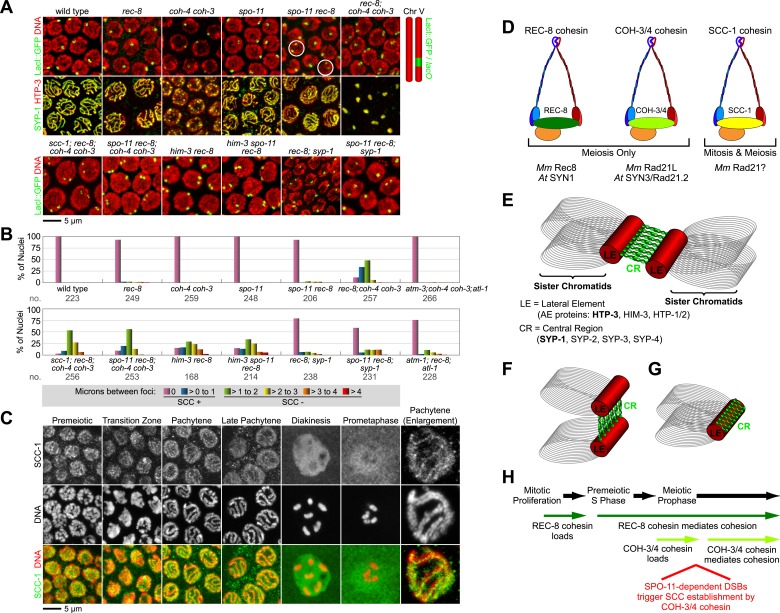
Cohesin-dependent and cohesin-independent SCC holds sisters together in pachytene nuclei. (**A**) Projections of confocal Z-sections through entire pachytene nuclei. A single LacI::GFP focus is detected in pachytene nuclei of wild-type, *coh-4 coh-3*, and *spo-11* mutant worms, indicating that sister chromatids are tethered by SCC. In contrast, sisters are separated in most pachytene nuclei of kleisin triple mutants but still remain close together, suggesting that residual SCC persists. Partial depletion of SCC-1 in kleisin triple mutant animals increases both the frequency of sister separation and the distance between sisters, demonstrating a meiotic role for SCC-1. Surprisingly, sisters could be resolved in only ∼10% of nuclei in *rec-8* and *spo-11 rec-8* worms (white circles). The robust synaptonemal complex (SC) assembly in *spo-11 rec-8* worms suggested that SC proteins may tether sister chromatids independently of cohesin. Indeed, disrupting the axial element (AE) protein HIM-3 severely compromised SCC in both *rec-8* and *spo-11 rec-8* mutants. Disrupting the central region (CR) protein SYP-1 had a lesser effect, suggesting that AE proteins can tether sisters together independently of CR proteins and cohesin. (**B**) Quantification of sister separation in pachytene nuclei. no. = number of nuclei scored. (**C**) Z-projected confocal images of wild-type gonads stained with DAPI and antibodies to SCC-1. Similar to REC-8, SCC-1 was detected in premeiotic nuclei and became enriched in axial structures of transition zone and pachytene nuclei. Nucleoplasmic staining obscured any chromosomal signal from pachytene exit until prometaphase; however, SCC-1 was undetectable following nuclear envelope breakdown in prometaphase, indicating that SCC-1 cohesin was removed from chromosomes during diplotene or diakinesis. (**D**) Similar sets of kleisins function during meiosis in *C. elegans,* mammals and plants. (**E**) A schematic of SC structure. Studies in worms have identified four components of the axial/lateral element, or LE (HTP-3, HIM-3, and the functionally redundant proteins HTP-1 and HTP-2) and four components of the CR (SYP-1, SYP-2, SYP-3, and SYP-4). (**F** and **G**) Two models of SC-dependent linkages between sisters. (**F**) CR proteins link AEs formed along each sister. (**G**) AE proteins hold sisters together independently of CRs. (**H**) REC-8 cohesin and COH-3/4 cohesin load onto chromosomes at different times and establish SCC by different mechanisms. **DOI:** http://dx.doi.org/10.7554/eLife.03467.013

In most pachytene nuclei of wild-type worms (>99%), *rec-8* single mutants (95%), and *coh-4 coh-3* double mutants (>99%), sisters were tethered (Figure 5A,B and Figure 5—figure supplement 1). In contrast, sisters were apart in most nuclei of meiotic kleisin triple mutants (55%), revealing that REC-8 and COH-3/4 cohesins establish SCC. However, the persistence of linkages in 45% of pachytene nuclei in kleisin triple mutants indicated that factors other than REC-8 and COH-3/4 cohesins can also tether sisters (Figure 5A,B and Figure 5—figure supplement 1).

**Figure 5—figure supplement 1.**
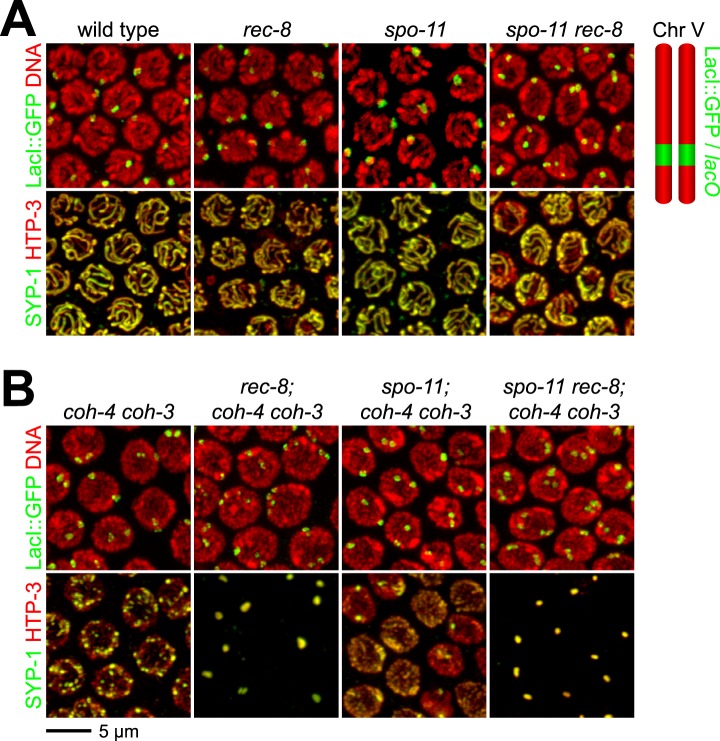
Synaptonemal complex (SC) proteins associate with pachytene chromosomes in *rec-8* and *spo-11 rec-8* animals, but they do not tether homologous chromosomes. Shown are confocal images of nuclei stained with LacI::GFP, which labels *lacO* arrays integrated into both chromosome V homologs (top panels) and antibodies to the axial/lateral element protein HTP-3 and the central region protein SYP-1 (bottom panels) (**A**) SC proteins associate with pachytene chromosomes of wild-type animals, *rec-8* and *spo-11* single mutants, and *spo-11 rec-8* double mutants. A single focus of LacI::GFP was detected in pachytene nuclei of wild-type and *spo-11* worms, as expected since homologs are fully synapsed in these animals. In contrast, two widely separated GFP foci were detected in *rec-8* and *spo-11 rec-8* mutants, suggesting that synapsis occurs between non-homologous chromosomes or sister chromatids rather than homologs. (**B**) SC proteins form polycomplexes in pachytene nuclei of *rec-8; coh-4 coh-3* triple mutants and *spo-11 rec-8; coh-4 coh-3* quadruple mutants. 3–4 LacI::GFP foci can be detected in most nuclei of these animals, consistent with a role for SC proteins in cohesin-independent SCC. **DOI:** http://dx.doi.org/10.7554/eLife.03467.014

**Figure 5—figure supplement 2.**
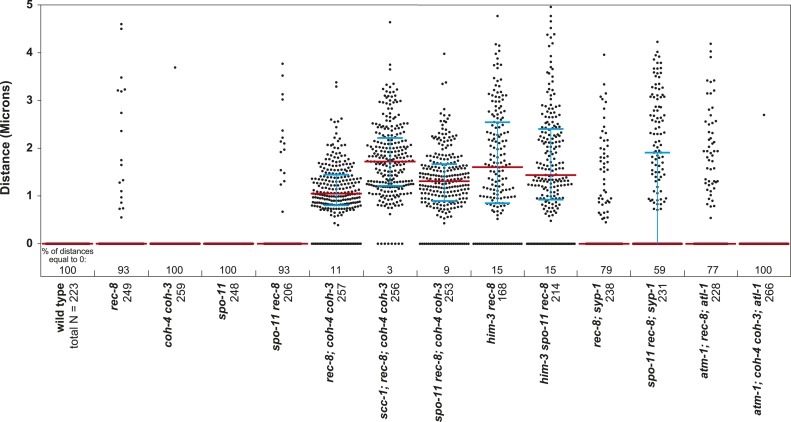
Beeswarm plots show individual distances between LacI::GFP foci in pachytene nuclei. Red horizontal lines indicate the median, and blue horizontal lines indicate the interquartile range. A distance of >1 micron indicates defective SCC. **DOI:** http://dx.doi.org/10.7554/eLife.03467.015

**Figure 5—figure supplement 3.**
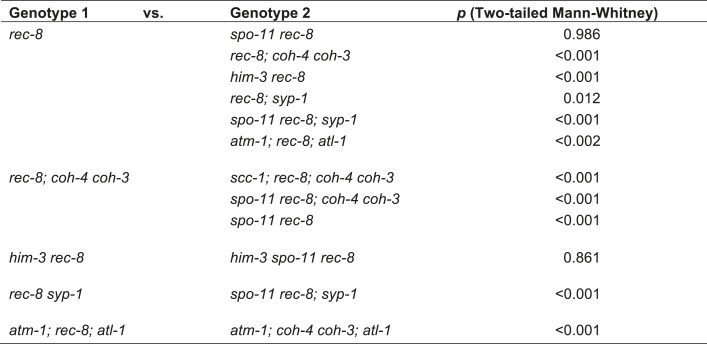
Table of significance values for distances between LacI::GFP foci in pachytene nuclei. **DOI:** http://dx.doi.org/10.7554/eLife.03467.016

We found that the *C. elegans* mitotic kleisin SCC-1, previously thought to function only during mitosis, contributes to cohesion in pachytene nuclei (Figure 5A,B and Figure 5—figure supplements 1 and 2). Since SCC-1 is required for mitotic proliferation of germline precursors, we used RNAi to partially deplete SCC-1 in kleisin triple mutants, then scored SCC in pachytene nuclei. Although LacI::GFP revealed extensive aneuploidy in premeiotic and transition zone nuclei of these animals, likely due to defective SCC during mitotic proliferation of the germline stem cells, only one or two LacI::GFP foci were detected in pachytene nuclei (Figure 5A,B, data not shown), indicating that nuclei with abnormal chromosomal number had not yet progressed into pachytene. Only 12% of pachytene nuclei exhibited cohesion in SCC-1-depleted kleisin triple mutants, indicating that SCC-1 cohesin can mediate REC-8 and COH-3/4-independent linkages (Figure 5 A,B and Figure 5—figure supplements 1 and 2). However, the finding that two LacI::GFP foci could be resolved in ∼89% of pachytene nuclei of kleisin triple mutants with wild-type levels of SCC-1 demonstrates that, unlike SCC mediated by REC-8 or COH-3/4 cohesin, SCC mediated by SCC-1 cohesin is insufficient to maintain the close association of sisters along their entire lengths. Thus, SCC-1 by itself is not likely to establish SCC in wild-type animals.

Consistent with the involvement of SCC-1 cohesin in tethering chromosomes during pachytene but not diakinesis, we observed that SCC-1 associates with axial structures of transition zone and pachytene nuclei of wild-type animals (Figure 5C). SCC-1 staining was detected between homologs, suggesting that SCC-1 cohesin associates with the meiotic chromosomal axis, similar to REC-8 and COH-3/4 cohesins. Diffuse nuclear staining obscured any chromosomal signal in diplotene/diakinesis nuclei; however, SCC-1 was undetectable on chromosomes following nuclear envelope breakdown, when the nucleoplasmic signal dissipated. Thus, any role played by SCC-1 in meiotic SCC of wild-type animals or kleisin triple mutants is likely to occur during pachytene, but not prometaphase I (See Discussion).

### Axial element protein HIM-3 can tether sisters independently of REC-8 and COH-3/4 cohesin

Unexpectedly, the absence of DSBs in *spo-11 rec-8* animals did not abrogate SCC in pachytene nuclei (6.8% lacking SCC), unlike in diakinesis nuclei (90% lacking SCC); however, we reasoned that axial element proteins and synaptonemal complex (SC) proteins (Figure 5E) might mediate the linkages that persist between sisters in pachytene nuclei of *spo-11 rec-8* mutants and thereby obscure the role of DSBs. In kleisin triple mutants, unlike in *spo-11 rec-8* mutants, SC proteins cannot account for the residual SCC, because SC assembly fails completely in these mutants, and all known SC proteins form nucleoplasmic aggregates called polycomplexes (Figure 5A, Figure 5—figure supplement 1B). However, SC assembly still occurs in both *spo-11 rec-8* double mutants and *rec-8* single mutants, likely between sister chromatids or non-homologous chromosomes, unlike in wild-type animals, and polycomplexes fail to form (Figure 5A, Figure 5—figure supplement 1A) (Pasierbek et al., 2001; Martinez-Perez et al., 2008; Severson et al., 2009; Rog and Dernburg, 2013). We therefore asked whether axial element (AE) proteins (Figure 5G) alone, or AE proteins bridged by SC central region (CR) proteins (Figure 5F) could tether sister chromatids (Figure 5A,B).

SCC was severely compromised in pachytene nuclei of animals lacking REC-8 and AE protein HIM-3. Sister chromatids were apart in ∼70% of pachytene nuclei of *him-3 rec-8* animals regardless of whether DSBs were made (Figure 5A,B and Figure 5—figure supplements 2 and 3), suggesting the involvement of AE proteins in tethering sister chromatids. The SCC defect did not result from a failure to form DSBs or to load COH-3/4 cohesin. Using RAD-51, a RecA homolog that binds to nascent recombination intermediates just after DSB formation, as a marker for DSBs, we found abundant RAD-51 foci in *him-3 rec-8* mutants in early and late pachytene (Figure 4B,C and Figure 4—figure supplement 1). Furthermore, COH-3/4 associated with meiotic chromosomes of *him-3 rec-8* animals, although the intensity of the COH-3/4 signal was less than that in wild-type animals due to defective synapsis and SCC (Figure 4A and legend and Figure 4—figure supplement 2). We therefore propose that HIM-3, or a protein that depends on HIM-3 for its loading, can tether sister chromatids during pachytene, independently of cohesin, thereby accounting, in part, for the SCC in *spo-ll rec-8* mutants.

### DSBs trigger COH-3/4 to become cohesive

We also discovered that disrupting the gene encoding the SC central region protein SYP-1 further reduces SCC in *spo-11 rec-8* animals (Figure 5A,B and Figure 5—figure supplements 2 and 3). In *spo-11 rec-8; syp-1* triple mutants, 36% of pachytene nuclei lacked SCC compared to 7% of nuclei in *spo-11 rec-8* double mutants and 71% of nuclei in *him-3 spo-11 rec-8,* indicating that SYP-1 assists in linking sisters independently of cohesin, but AE protein HIM-3 plays a more prominent role.

The more minor involvement of SYP-1 in tethering sister chromatids provided the opportunity to assess whether DSBs trigger COH-3/4-dependent SCC. We found that the frequency of sister separation in *spo-11 rec-8; syp-1* triple mutants (36%) was greater than in *rec-8; syp-1* double mutants (14%) (p < 0.001) indicating that DSBs play an important role in triggering COH-3/4-dependent SCC (Figure 5A,B and Figure 5—figure supplements 2 and 3).

Further indication of the key role played by DSBs in establishing COH-3/4-dependent SCC came from analysis of pachytene nuclei in *rec-8* single and *coh-4 coh-3* double mutants that were also defective in the ATM/ATR signaling cascade. If DSBs are important for triggering COH-3/4 to be cohesive, the *rec-8* mutants should exhibit greater sister separation than the *coh-4 coh-3* mutants when this signaling cascade is defective. Indeed, while 0% of *atm-1; coh-4 coh-3; atl-1* mutants showed sister separation in pachytene nuclei, 21% of *atm-1; rec-8; atl-1* mutants exhibited separation (p < 0.001) (Figure 5B and Figure 5—figure supplements 2 and 3).

These results indicate that DSBs trigger COH-3/4 to become cohesive. The participation of AE and SC components in tethering sisters during pachytene in *spo-11 rec-8* mutants made it difficult to discover this role. Although CR proteins are unlikely to tether sisters during wild-type meiosis, AE-dependent linkages between sisters may be a normal feature of meiosis (See Discussion).

### Cohesin is required for the formation of meiotic DSBs

As expected, we found chromosome fragments and fusions in 100% of nuclei in animals with severely compromised REC-8- and COH-3/4-dependent SCC (e.g. *him-3 rec-8* and *atm-1; rec-8; atl-1* mutants) as a consequence of defective DSB repair (Figure 3F; Figure 4D). Unexpectedly, we found very few RAD-51 foci (Figure 4B,C) and virtually no chromosome fragments or fusions (Figure 3D and Figure 4D) in all 30 of the gonads examined from meiotic kleisin triple mutants, suggesting a nearly complete absence of early DSB repair intermediates and little or no defective DSB repair. We conclude that cohesin, but not SCC per se, is necessary for the timely formation of RAD-51 foci, and likely DSBs. Dependence of DSB formation on REC-8 and COH-3/4 could reflect either a direct requirement for cohesin in the formation of SPO-11-dependent DSBs or instead the known requirement for REC-8 and COH-3/4 cohesin in loading HTP-3 (Severson et al., 2009), which is essential for DSB formation (Goodyer et al., 2008).

### CO recombination triggers selective removal of REC-8 and COH-3/4 cohesins from reciprocal domains to facilitate homolog and sister separation

**Figure 6.**
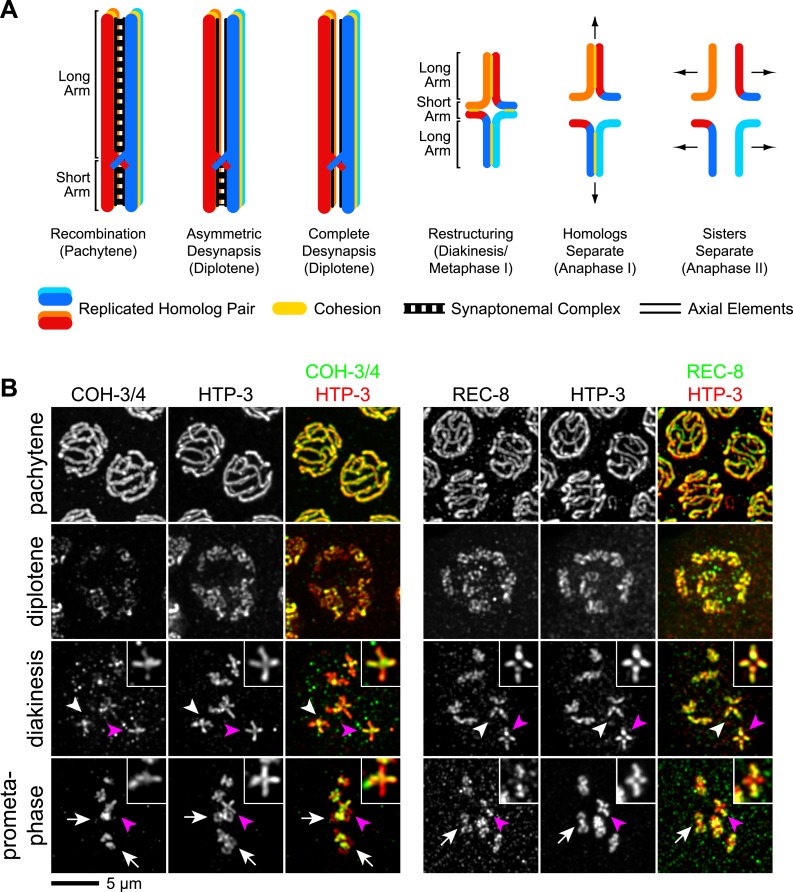
CO recombination triggers removal of REC-8 and COH-3/4 cohesins from reciprocal domains in late prophase/prometaphase of meiosis I. (**A**) In worms, CO position determines where SCC will be removed in anaphase I. A single, asymmetrically positioned CO forms between each homolog pair in pachytene, dividing the homologs into long and short arms. In diplotene, each homolog pair is restructured around the CO to form a cruciform bivalent. At anaphase I, SCC is released at the short arm to allow homologs to separate. SCC persists at the long arm until anaphase II. (**B**) Confocal micrographs showing that REC-8 and COH-3/4 adopt complementary patterns on meiotic chromosomes by metaphase. In pachytene, REC-8 and COH-3/4 overlap with HTP-3 along the entire meiotic axis. In diplotene, HTP-3 and REC-8 persist along the length of the axis, but COH-3/4 staining diminishes at long arms. By diakinesis, COH-3/4 levels are substantially reduced at long arms but not at short arms. In contrast, REC-8 levels usually remain equal at long and short arms until late diakinesis or prometaphase. Diakinesis nuclei shown are from the third oldest oocyte. In prometaphase/metaphase I, REC-8 and COH-3/4 occupy reciprocal domains. REC-8 is reduced or undetectable at short arms, while COH-3/4 is detectable only at short arms. Arrowheads indicate bivalents viewed from the ‘front’, that is with both long and short arms in the image plane. In these bivalents, HTP-3 staining is cruciform and long and short arms can usually be distinguished by their relative lengths. Pink arrowheads indicate the bivalent shown at higher magnification in the inset. Arrows indicate bivalents viewed from the ‘side’, that is with short arms perpendicular to the image plane. In these bivalents, HTP-3 staining resembles a ‘figure 8’, with two loops of uniform staining (the long arms) meeting at a region of more intense staining (the short arms). **DOI:** http://dx.doi.org/10.7554/eLife.03467.017

In *C. elegans*, a single, asymmetrically positioned CO forms between each homolog pair. The CO divides the pair into long and short arms (Barnes et al., 1995; Albertson et al., 1997; Nabeshima et al., 2005) (Figure 6A). The holocentric chromosomes of *C. elegans* lack a localized centromere; however, the long arms share features with centromeres of monocentric chromosomes during meiosis. Co-orientation occurs at the long arms to ensure that the two sister chromatids interact with microtubules from the same spindle pole, and SCC is maintained at the long arms until anaphase II to keep sisters together. Cohesion at the short arms holds homologs together, and SCC release at short arms in anaphase I triggers disjunction of homologous chromosomes. In other words, the CO site, not a centromere, defines a region of each homolog in which co-orientation is implemented and SCC persists until anaphase II (Reviewed in Schvarzstein et al., 2010).

During diplotene and diakinesis, a condensin-dependent process restructures the short and long arms of each recombined homolog pair around the CO to form a cruciform (Figure 6A; Chan et al., 2004). We found that during this reorganization, COH-3/4 was removed from the long arm and became restricted to the short arm by prometaphase (Figure 6B). The opposite pattern of removal was noted for REC-8 (e.g., Figure 3E,F in de Carvalho et al., 2008; Supplemental Figure 1D in Harper et al., 2011; Figure 5 in Rogers et al., 2002). We confirmed that REC-8 is progressively removed from the short arm of diakinesis bivalents and often becomes undetectable by metaphase I (Figure 6B). The redistribution of COH-3/4 precedes that of REC-8, which is usually not apparent until late diakinesis or prometaphase I (Figure 6B). Thus, the kleisin subunit determines both when and where a cohesin complex will be removed from chromosomes during late prophase and prometaphase.

**Figure 7.**
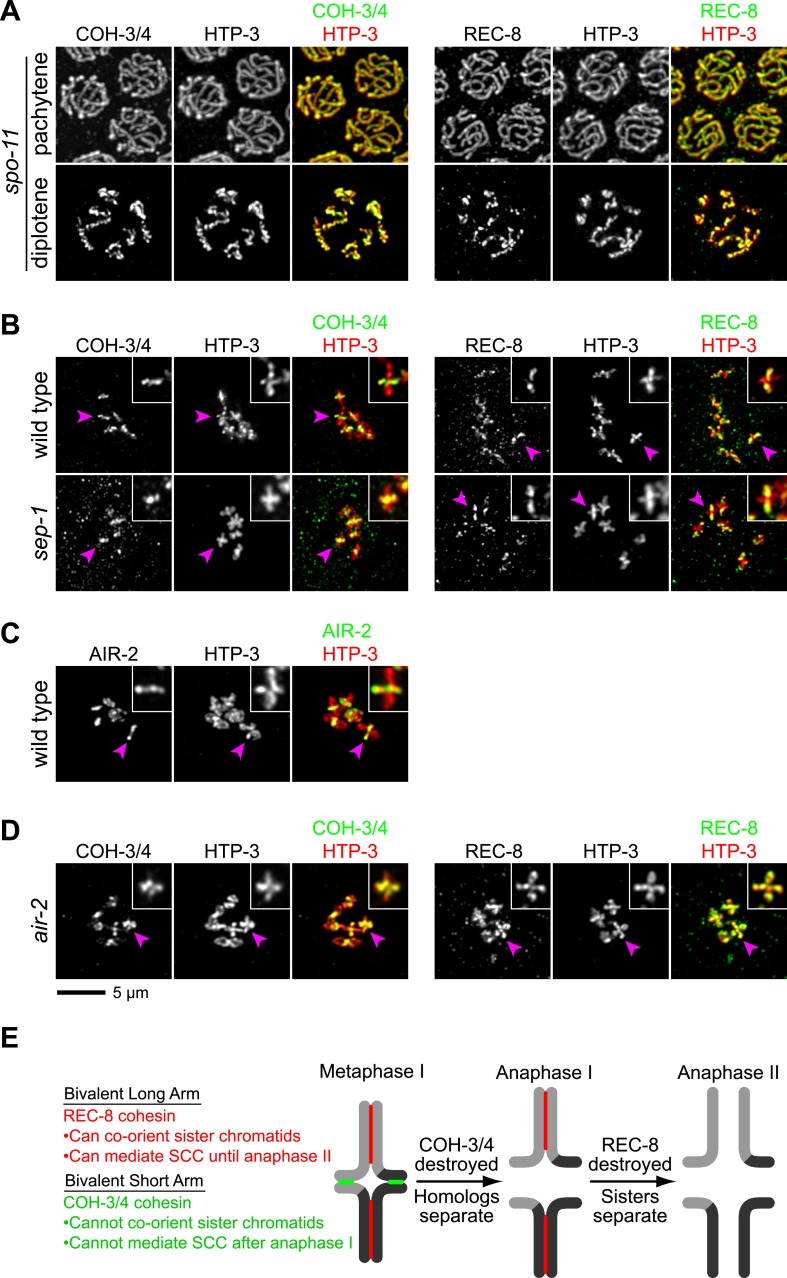
CO recombination triggers separase-independent removal of REC-8 and COH-3/4 from complementary chromosomal territories. (**A**–**D**) Projected images of entire nuclei in pachytene and diplotene (**A**) or diakinesis (**B**–**D**). (**A**) In *spo-11* mutants, CO recombination fails and REC-8 and COH-3/4 are present along the length of meiotic axes in pachytene and diplotene nuclei. In diakinesis, both kleisins are detected at the mid-univalent (Figure 7—figure supplement 1). (**B**) Depletion of the separase ortholog *sep-1* does not impede removal of REC-8 or COH-3/4. (**C**) AIR-2 associates with short arms of diakinesis bivalents. (**D**) In *air-2(RNAi)* animals, REC-8 persists on both long and short arms of prometaphase bivalents, indicating that AIR-2 is required for removal of REC-8 from short arms. COH-3/4 still persists at the midbivalent, indicating that AIR-2 is not required for removal of COH-3/4 from long arms or maintenance of COH-3/4 at short arms. (**E**) A model demonstrating how establishing reciprocal domains of REC-8 cohesin and COH-3/4 cohesin could facilitate sequential separation of homologs and then sisters. REC-8 cohesin (red) can co-orient sister chromatids and mediate SCC that persists until anaphase II. COH-3/4 cohesin (green) cannot. Restricting REC-8 cohesin to long arms would ensure that co-orientation and persistent SCC occur only in this domain. Co-orientation of long arms would ensure that sister chromatids are pulled to the same spindle pole in anaphase I following proteolytic cleavage of COH-3/4. Proteolysis of REC-8 in meiosis II would allow sisters to separate. **DOI:** http://dx.doi.org/10.7554/eLife.03467.018

The partitioning of REC-8 and COH-3/4 into reciprocal domains that flank the CO site suggests that CO recombination triggers removal of REC-8 and COH-3/4 cohesins from complementary regions of the bivalent. Indeed, REC-8 and COH-3/4 persist along the entire axis of desynapsing chromosomes in diplotene nuclei of *spo-11* mutants (Figure 7A), and both kleisins associate with the midunivalent of the detached homologs in diakinesis nuclei (Figure 7—figure supplement 1).

**Figure 7—figure supplement 1.**
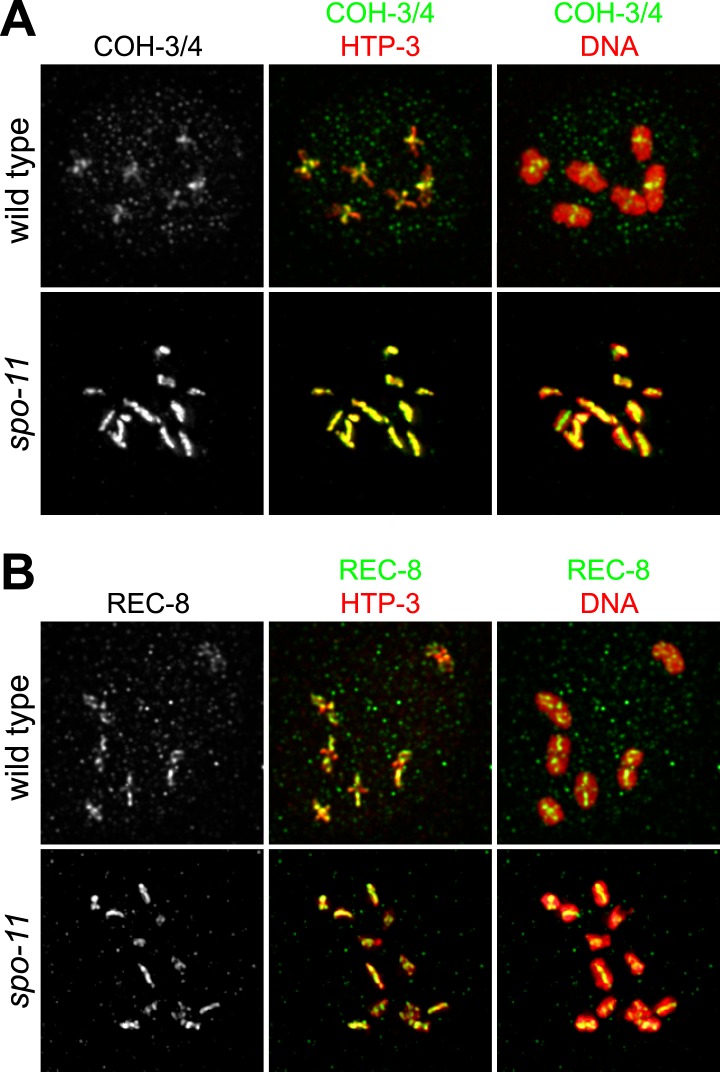
SPO-11-dependent CO recombination triggers the removal of REC-8 and COH-3/4 from complementary domains. Projected confocal micrographs show the distribution of COH-3/4 and REC-8 on diakinesis bivalents in wild-type worms and univalents of *spo-11* mutants. (**A**) COH-3/4 become substantially reduced or undetectable at the long arms of wild-type bivalents by diakinesis, but persists at high levels at short arms. In contrast, the AE protein HTP-3 is present at uniform levels at both long and short arms. (**B**) In contrast, REC-8 becomes reduced at short arms but persists at high levels at long arms. CO recombination fails in *spo-11* mutants, and homologs remain apart as discrete univalents. COH-3/4 (**A**) and REC-8 (**B**) both associate with univalents in diakinesis nuclei of *spo-11* mutants. **DOI:** http://dx.doi.org/10.7554/eLife.03467.019

During mitosis in many organisms, cohesin complexes are removed from chromosomes by two pathways (Nasmyth and Haering, 2009). In prophase, cohesin complexes are removed from chromosome arms via the prophase pathway, a non-proteolytic pathway that involves phosphorylation by Polo and Aurora B kinases. At anaphase onset, cohesin is removed from centromeres by proteolysis of the kleisin by the cysteine protease separase (Buonomo et al., 2000; Uhlmann et al., 2000). The timing of removal of REC-8 and COH-3/4 suggested that cohesin complexes are triggered to dissociate from chromosomes in prophase, independently of separase. Consistent with this, removal of REC-8 from the short arm did not require the worm separase homolog SEP-1 (Figure 7B) but did require the Aurora B kinase AIR-2, which accumulates at the short arm in late diakinesis, prior to the reduction of REC-8 in this region (Figure 7C,D; Rogers et al., 2002). These data are consistent with removal of REC-8 cohesin from the short arm by a meiotic prophase pathway. Dissociation of COH-3/4 from the long arm was also independent of SEP-1. However, neither removal of COH-3/4 from the long arm nor maintenance of COH-3/4 at the short arm required AIR-2 (Figure 7D). These data are consistent with our finding that COH-3/4 begins to disappear from the long arm in diplotene, well before the accumulation of AIR-2 at the short arm. Thus, the kleisin determines not only when and where, but also how a cohesin complex is removed from chromosomes. Kleisin-specified cohesin removal in late prophase could promote the stepwise separation of homologs and sisters, a model consistent with the mutant phenotypes of *rec-8* single and *coh-4 coh-3* double mutants.

## Discussion

### Kleisin subunits specify meiotic cohesin function

We showed that multiple, functionally distinct cohesin complexes mediate sister chromatid cohesion during meiosis. The cohesins differ in their α-kleisin subunit, and the kleisin influences nearly all aspects of meiotic cohesin function: the mechanisms for loading cohesins onto chromosomes, for triggering DNA-bound cohesins to become cohesive, and for releasing cohesins in a temporal- and location-specific manner during prophase I. Our findings establish a new model for cohesin function in meiosis: the choreographed actions of multiple cohesins, endowed with specialized functions by their kleisins, underlie the stepwise separation of homologs and sisters essential for the reduction of genome copy number.

### DSB-induced tethering of sister chromatids is an essential feature of meiosis

Our work demonstrated a critical and unexpected role for DSBs in triggering meiotic SCC. The importance of DSBs in inducing SCC was shown previously only in proliferating yeast cells that suffered DNA damage during G2/M. Because Rec8 cohesin failed to establish DSB-induced cohesion when replacing Scc1 in mitotic cells, the DSBs used to initiate CO recombination during meiosis were presumed not to trigger SCC (Heidinger-Pauli et al., 2008). To the contrary, we found meiotic DSBs to be the essential trigger that induces COH-3/4 cohesin to tether sisters. Mutants deficient in SPO-11 failed to form DSBs and failed to generate COH-3/4–dependent cohesion unless subjected to ionizing radiation. Thus, DSB-induced SCC is an essential, conserved process that functions not only in proliferating yeast cells suffering DNA damage, but also in nematode germ cells undergoing normal gametogenesis.

Unexpectedly, although SCC was severely disrupted in diakinesis nuclei of *spo-11 rec-8* mutants, indicating that DSBs are essential for tethering sister chromatids, the sisters were together in most pachytene nuclei of *spo-11 rec-8* mutants, raising the question of whether DSBs are essential for establishing COH-3/4-mediated cohesion. Several lines of evidence demonstrate a requirement for DSBs in triggering COH-3/4-mediated SCC. First, COH-3/4 was not detected in PCNA-positive nuclei, suggesting that a replication-independent mechanism initiates the process by which COH-3/4 cohesin becomes cohesive. DSBs are the only known trigger for establishing SCC outside of S phase. Second, the AE protein HIM-3 can tether sisters in pachytene nuclei independently of REC-8 in both *rec-8* single and *spo-11 rec-8* double mutants, thereby obscuring the role of DSBs in establishing COH-3/4-mediated SCC. Third, synaptonemal complexes form between sisters in *rec-8* and *spo-11 rec-8* animals. Disrupting DSB formation in *rec-8* mutants lacking the SC central region protein SYP-1 (*i.e., spo-11 rec-8; syp-1* triple mutants) increased the frequency of sister separation in pachytene nuclei relative to that of *rec-8; syp-1* double mutants proficient in DSB formation. This result strengthens the view that DSBs are required to establish SCC, but the requirement is partially masked by sister linkages mediated by SC proteins. Finally, sister separation is greater in pachytene nuclei of *rec-8* mutants with a defective ATM/ATR signaling pathway than in *rec-8* mutants with a functional pathway.

In *Saccharomyces cerevisiae*, DNA damage in G2/M activates ATM and Chk1 kinases, leading to Chk1-dependent phosphorylation of Scc1 on serine 83. Unphosphorylatable S83A mutants fail to establish DI-SCC, while phosphomimetic S83D mutants establish DI-SCC independently of Chk1, showing the importance of S83 phosphorylation in inducing cohesion in response to DNA damage (Heidinger-Pauli et al., 2009, 2008). Yeast Rec8 lacks an equivalent Chk1 consensus site, explaining its failure to establish DI-SCC. SPO-11-dependent DSBs appear to activate a similar signaling cascade to trigger meiotic DI-SCC through COH-3/4 cohesin in *C. elegans*. This cascade requires ATM, ATR, and CHK-2 and likely culminates in kleisin phosphorylation. Because *rec-8; chk-2* mutants exhibited reduced COH-3/4 staining and complete failure to tether sisters, CHK-2 may function in regulating cohesin loading as well as in triggering sister linkages. To assess whether the loading and SCC establishment defects are separable, the predicted CHK kinase phosphosites in COH-3/4 are being changed to alanine. S81 and/or T82 in COH-3/4 are the most plausible sequence homologs of S83 in yeast Scc1, but COH-3 and COH-4 also have four sites with a perfect match to the Chk kinase consensus sequence that might serve as targets of CHK-2 phosphorylation required for SCC establishment by COH-3/4 cohesin.

### Meiotic cohesin complexes function in cohesion-independent processes

The crucial role of DSBs in establishing COH-3/4-dependent cohesion allowed us to answer a longstanding question: does SC assembly require only the chromosomal binding of cohesin or also the conversion of bound cohesin into a cohesive state? The strikingly different SC assembly defects in mutants that retain COH-3/4 binding but lack both REC-8- and COH-3/4-mediated cohesion (*spo-11 rec-8)* compared to mutants that lack COH-3/4 binding as well as REC-8- and COH-3/4-dependent cohesion (*rec-8; coh-4 coh-3*) revealed the answer. Robust SC assembled along chromosomes of *spo-11 rec-8* mutants, between sister chromatids or non-homologous chromosomes, but SC assembly failed on chromosomes of kleisin triple mutants. Thus, SC assembly requires COH-3/4 binding but not its conversion to a cohesive state, revealing that cohesin functions in cohesion-independent processes. These data also demonstrated that SC assembly is more sensitive to disruption of COH-3/4 cohesin than REC-8 cohesin, since we observed severe SC structural defects in *coh-4 coh-3* double mutants but not in *rec-8* single mutants (See also Severson et al., 2009). Thus, the kleisin determines the role of cohesins in synaptonemal complex (SC) assembly.

### REC-8 and COH-3/4-independent mechanisms can tether sister chromatids during early meiosis

It was previously believed that meiosis-specific cohesin complexes were both necessary and sufficient to establish and maintain the linkages that tether sisters during gametogenesis. Eliminating REC-8- and COH-3/4-dependent SCC enabled us to discover the participation of mitotic kleisins and axial proteins in chromosome tethering during meiosis. In diakinesis nuclei, REC-8 and COH-3/4 cohesins are indispensable for holding sister chromatids together. In contrast, although REC-8 and COH-3/4 are critical for establishing meiotic SCC, weak linkages can occur between sisters in pachytene nuclei of mutants lacking the three meiotic kleisins. These linkages are mediated by SCC-1 cohesin, previously believed to function only during mitosis.

SCC-1 associates with meiotic chromosomal axes in transition-zone and pachytene nuclei of wild-type animals, suggesting that SCC-1 has the capacity to mediate SCC during wild-type meiosis. However, the role of SCC-1 during wild-type meiosis is not easy to determine. No obvious meiotic phenotypes were detected following depletion of SCC-1 in wild-type animals (data not shown); but this analysis was limited by the need to use partial loss-of-function conditions due to the essential role of SCC-1 in mitotic proliferation of germline precursors.

Mitotic kleisins also associate with meiotic chromosomes of budding yeast and mice (Klein et al., 1999; Xu et al., 2004; Ishiguro et al., 2011; Lee and Hirano, 2011). The Rad54 paralog Tid1 is necessary to remove Scc1-dependent linkages from yeast meiotic chromosomes to facilitate normal chromosome segregation (Kateneva et al., 2005), indicating that Scc1 cohesin can tether sisters during meiosis. Meiotic roles for mammalian Rad21/Scc1 have not yet been defined. Nevertheless, the association of Scc1 orthologs with meiotic chromosomes of widely diverged species suggests they play important roles in gametogenesis.

Although SCC-1 can tether sisters in pachytene nuclei of *rec-8; coh-4 coh-3* mutants, SC proteins do not associate with meiotic chromosomes. Thus, unlike REC-8 and COH-3/4, SCC-1 appears unable to promote even partial SC assembly. These data demonstrate that establishment of cohesion between sisters is insufficient to promote the formation of SC, consistent with our finding that SC assembly requires chromosomally bound COH-3/4 cohesin, but not conversion of COH-3/4 cohesin into a cohesive state.

We also found that axial element (AE) proteins can mediate cohesin-independent linkages between sister chromatids when *rec-8-* and DSB-dependent cohesion fail to occur. That is, HIM-3 tethers sisters independently of cohesin in *spo-11 rec-8* mutants, which have chromosome-bound COH-3/4 that is not cohesive. The HIM-3-dependent linkages are not mediated by an SC-like structure, given that disrupting the CR protein SYP-1 only partially weakened SCC in *spo-11 rec-8* double mutants, while mutations in *him-3* severely abrogated SCC. HIM-3-dependent linkages between sisters may not be essential in animals with wild-type REC-8, because REC-8-dependent SCC established during premeiotic DNA replication is likely sufficient to tether sisters until anaphase II (Severson et al., 2009). However, HIM-3-dependent linkages may prevent newly replicated sister chromatids from drifting apart in *rec-8* mutants before DSBs trigger COH-3/4 to become cohesive, thus explaining the severe SCC defects observed in *him-3 rec-8* double mutants with wild-type *spo-11*.

Single molecule experiments have shown that purified Hop1, a HORMA domain protein that is the yeast ortholog of HIM-3, HTP-3, and HTP-1/2, can mediate *trans* interactions that bridge linear double-stranded DNA molecules and promote their restructuring and compaction (Khan et al., 2012). These interactions were proposed to facilitate pairing or synapsis of homologous chromosomes, but our data suggest a different role: HORMA-domain AE proteins such as HIM-3 may directly tether sister chromatids independently of cohesin in wild-type animals, as we have shown for cohesion-defective mutants.

The mutant analysis described here reveals the unexpected molecular complexity of meiotic SCC and suggests that the linkages that tether sisters in the germline are not formed synchronously during DNA replication, as occurs during mitosis, but rather through the sequential action of proteins that bind to chromosomes at temporally distinct times and generate cohesion by different mechanisms. Our data support the following model. REC-8 cohesin mediates the first wave of SCC establishment. SCC-1 may function together with REC-8 during that process. Both REC-8 and SCC-1 are present in all premeiotic nuclei and likely bind to chromatids prior to premeiotic S phase and become cohesive during DNA replication, a pattern similar to that described for cohesin complexes in mitotically proliferating cells. HIM-3 may mediate a second wave of cohesion, but through a mechanism distinct from that of cohesin. HIM-3 is expressed and loads onto chromosomes in leptotene, at the onset of meiosis (Zetka et al., 1999; MacQueen et al., 2002), and our data suggest that HIM-3 tethers sisters independently of REC-8 and COH-3/4 cohesin. More definitive evidence for the involvement of HIM-3 in mediating cohesion will require a separation-of-function allele that disrupts SCC but not SC assembly or crossover recombination. A final wave of SCC establishment is mediated by COH-3/4 cohesin, which loads onto chromosomes at the onset of meiosis but is not triggered to become cohesive until the formation of SPO-11-dependent DSBs during leptotene and/or zygotene. Sequential mutational analysis in kleisin-defective animals enabled us to strip away the layers of chromosome tethering that are mediated by mitotic kleisin SCC-1, axial protein HIM-3, and SC to uncover the central roles of DSBs, REC-8, and COH-3/4 in establishing SCC.

Our data also suggest that the removal of linkages between sisters occurs in stages. In diplotene (our unpublished data) and diakinesis nuclei, sisters are held together by REC-8 and COH-3/4 cohesin alone, indicating that the HIM-3 and SCC-1-dependent linkages we demonstrated in pachytene are transient and are removed during late pachytene or early diplotene. In contrast, SCC mediated by COH-3/4 and REC-8 persists until anaphase I and II, respectively (see below).

### A model for the role of distinct cohesin complexes in reducing genome copy number during meiosis

Prior to this study and our earlier demonstration that COH-3/4 and REC-8 mediate meiotic SCC (Severson et al., 2009), the triggering and release of meiotic cohesion were thought to depend entirely on Rec8 cohesin. All models of eukaryotic meiotic chromosome segregation asserted that the stepwise cleavage of Rec8 by separase initiated the successive separation of homologs and sisters, and thus the production of haploid gametes.

In organisms with monocentric chromosomes, cleavage of Rec8 along chromosome arms was proposed to trigger homolog separation in meiosis I, while cleavage of Rec8 at centromeres was proposed to allow sister separation in meiosis II (Klein et al., 1999; Watanabe and Nurse, 1999; Buonomo et al., 2000; Watanabe, 2004; Kudo et al., 2006). In the holocentric nematode *C. elegans,* where the asymmetric CO site rather than the centromere defines the bivalent short and long arms, cleavage of REC-8 at the short arm was believed to elicit homolog segregation in meiosis I, while cleavage of REC-8 at the bivalent long arm was believed to cause sister separation in meiosis II (Pasierbek et al., 2001; Siomos et al., 2001; Kaitna et al., 2002; Rogers et al., 2002).

Consistent with this model, prior *C. elegans* studies identified the Aurora B kinase AIR-2 as a key factor that regulates SCC release, and thus chromosome segregation, during meiosis and mitosis. In meiosis I, AIR-2 accumulates at bivalent short arms and is essential for homolog separation (Kaitna et al., 2002; Rogers et al., 2002). In meiosis II, AIR-2 accumulates between sisters and is required for sister separation. AIR-2 can phosphorylate REC-8 in vitro (Rogers et al., 2002). Thus, the distribution of AIR-2 predicts where SCC will be released at anaphase in meiosis I and II.

AIR-2 is prevented from accumulating at the long arms during meiosis I by the partially redundant AE proteins HTP-1 and HTP-2 (HTP-1/2) and the novel, *Caenorhabditis-*specific protein LAB-1 (de Carvalho et al., 2008; Kaitna et al., 2002; Martinez-Perez et al., 2008; Rogers et al., 2002). HTP-1/2 and LAB-1 accumulate along the entire length of meiotic chromosomal axes in early pachytene, but CO recombination triggers their removal from short arms and thereby allows the accumulation of AIR-2 at the short arms. HTP-1/2 and LAB-1 are undetectable on chromosomes after anaphase I; consequently, AIR-2 accumulates between sisters in meiosis II. In *htp-1 htp-2* and *lab-1* mutants, AIR-2 associates with both long and short arms. As a consequence, sister chromatids separate prematurely during anaphase I.

The correlation between the presence of AIR-2 and the release of SCC during meiosis of wild-type animals and *htp-1 htp-2* and *lab-1* mutants, together with the finding that AIR-2 can phosphorylate REC-8 in vitro, led to the model that AIR-2 induces the stepwise separation of homologs and sisters by phosphorylating REC-8, first at the short arm to trigger separase-dependent cleavage in anaphase I, then at the long arm to trigger separase-mediated cleavage in anaphase II (Kaitna et al., 2002; Rogers et al., 2002). However, it has never been determined whether AIR-2-dependent phosphorylation is required for separase to cleave REC-8 or whether cleavage of REC-8 by separase is required for homolog separation at anaphase I. Thus, this model has never been put to a rigorous test.

Together, our discovery of COH-3/4 in *C. elegans* and our demonstration that CO recombination consistently triggers separase-independent removal of REC-8 and COH-3/4 cohesins from reciprocal domains of meiosis I bivalents indicate that prior models are insufficient to explain how linkages are removed between sister chromatids to permit the separation of homologs and then sisters. Any model of *C. elegans* meiotic chromosome segregation must incorporate not only the removal of REC-8 cohesin by separase, but also the separase-dependent removal of COH-3/4 cohesin and the role of separase-independent cohesin removal that occurs in late prophase.

Our finding that different mechanisms trigger separase-independent removal of REC-8 from short arms and COH-3/4 from long arms indicates that AIR-2 is not the sole factor to direct the stepwise separation of homologs and sisters. REC-8 persists at high levels at the long arm but becomes markedly reduced and often undetectable at the short arm (our work and de Carvalho et al., 2008; Rogers et al., 2002; Harper et al., 2011). Removal of REC-8 from the short arm begins in late diakinesis or prometaphase and requires AIR-2. In contrast, COH-3/4 cohesin is removed from the long arm of wild-type animals beginning in diplotene, prior to AIR-2 accumulation at the midbivalent and coincident with the removal of SC from the long arm (Nabeshima et al., 2005). Both removal of COH-3/4 from the long arm and persistence of COH-3/4 at the short arm are independent of AIR-2 function. The factors that restrict COH-3/4 cohesin to the short arm are not known, but could include HTP-1/2, LAB-1, and PP1; however, if these factors regulate the distribution of COH-3/4 cohesin, they must do so in parallel with their regulation of AIR-2.

We propose two models for how separase-independent cohesin removal in prophase could promote the separation of homologs before sisters in *C. elegans*. In the first model, REC-8 cohesin is eliminated from the short arm by an AIR-2-dependent mechanism that is independent of kleisin proteolysis, allowing homolog disjunction at anaphase I to be triggered by separase-dependent cleavage of COH-3/4 (Figure 7E). The separase-independent partitioning of REC-8 and COH-3/4 cohesins into reciprocal domains could explain why factors like Mei-S332/Shugoshin, which protect centromeric cohesin from separase-mediated cleavage during anaphase I in monocentric organisms, are not required in *C. elegans* (de Carvalho et al., 2008; Severson et al., 2009).

In the second model, selective removal of REC-8 from the short arm does not determine the timing of separation for homologs vs sisters, but rather restricts co-orientation to the long arm. Once homologs have made proper attachments to microtubules and aligned on the metaphase plate, separase-dependent cleavage of COH-3/4 and any REC-8 remaining at the short arm would allow homologs to segregate toward opposite poles. Because REC-8 and COH-3/4 are both required for CO recombination, and hence the formation of bivalents with differentiated long and short arms, a direct test of these models will require versions of REC-8 and COH-3 or COH-4 that can be removed from chromosomes after COs have formed.

### A new model for meiotic cohesin function in higher eukaryotes

Our work reveals the unexpected degree to which kleisin variants influence virtually all facets of meiotic cohesin function. It establishes a new model for cohesin function during gametogenesis in higher eukaryotes. The orchestrated actions of multiple cohesins, endowed with specialized functions by their kleisins, reduce genome copy number to produce haploid gametes. The kleisin determines the mechanisms by which cohesin loads onto meiotic chromosomes, establishes SCC, and is removed from chromosomes prior to proteolytic cleavage by separase at anaphase I. Plants and mammals require similar sets of meiotic kleisins as those in *C. elegans*, demonstrating the widely conserved involvement of multiple kleisins in gametogenesis and highlighting the importance of understanding the mechanisms by which kleisins influence cohesin function. Our work represents a major stride toward achieving that goal.

The phenotypes of kleisin-deficient mice and the published localization patterns of mammalian kleisins suggest that the models established for *C. elegans* will apply to mammals. For example, as in *C. elegans* (Figure 5A and Severson et al., 2009), SC proteins in mice assemble between sister chromatids in *Rec8* single mutants but not in *Rad21L Rec8* double kleisin mutants, demonstrating the involvement of multiple kleisins in SC assembly (Llano et al., 2012; Ishiguro et al., 2014). Moreover, the idea that factors other than known meiosis-specific cohesin complexes contribute to SCC during spermatogenesis is suggested by the persistence of partial cohesion in meiotic nuclei of mouse *Rad21L Rec8* double mutants (Llano et al., 2012; Ishiguro et al., 2014). The mitotic kleisin RAD21 appears to associate with meiotic chromosomes of both wild-type and *Rad21L Rec8* mutants, suggesting that ‘mitotic’ cohesin might tether sisters during gametogenesis in mammals, as in worms (Prieto et al., 2002; Parra et al., 2004; Xu et al., 2004; Ishiguro et al., 2011; Lee and Hirano, 2011; Llano et al., 2012).

The published data for mice are also consistent with our findings that the kleisin determines the mechanisms of cohesin loading and SCC establishment. High levels of REC8 were detected in premeiotic mouse nuclei, consistent with REC8 cohesin becoming cohesive during DNA replication (Eijpe et al., 2003; Ishiguro et al., 2014). In contrast, RAD21L staining was faint in PCNA-positive premeiotic testicular cells but greatly increased on meiotic chromosomes during leptotene and zygotene (Herran et al., 2011; Lee and Hirano, 2011; Ishiguro et al., 2014), indicating that substantial amounts of RAD21L cohesin load after meiotic entry. Thus, different mechanisms may promote the premeiotic loading of REC8 cohesin and the post-replicative loading of RAD21L cohesin, a model consistent with a role for programmed meiotic DSBs in triggering replication-independent SCC establishment by RAD21L cohesin.

Indeed, a *Spo11* disruption exacerbated the SCC defects of *Rec8* knockout mice (Ishiguro et al., 2014). However, the partial sister separation observed in *Spo11 Rec8* double mutants could have resulted from defective SC assembly rather than a failure to establish DSB-induced SCC, since DSBs promote formation of SC between homologs of wild-type animals and between sister chromatids of *Rec8* mutant mice (Romanienko and Camerini-Otero, 2000; Xu et al., 2005; Ishiguro et al., 2014). On the other hand, while DSBs may be critical for RAD21L cohesin to establish SCC, the sisters may have remained tethered in *Spo11 Rec8* mutants by RAD21 cohesin, thereby obscuring the essential role of DI-SCC. Thus, establishing whether DSBs are essential for mammalian meiosis will require an assessment of whether AE and/or SC CR proteins can tether sisters in mice, as they do in worms, and whether RAD21 cohesin contributes to meiotic SCC.

Finally, studies hint that the kleisin subunit of mammalian cohesin complexes may determine whether a complex will be removed during late prophase I via a separase-independent mechanism. Although REC8 persists at high levels at centromeres and chromosome arms until anaphase I in mouse spermatocytes and oocytes, RAD21L and RAD21 proteins progressively diminish in abundance during late prophase at chromosome arms of spermatocytes and at arms and centromeres of oocytes. (Prieto et al., 2002, 2004; Lee et al., 2003; Parra et al., 2004; Tachibana-Konwalski et al., 2010; Ishiguro et al., 2011; Lee and Hirano, 2011).

Understanding the mechanisms by which the kleisin subunit influences cohesin function to reduce genome copy number during meiosis of plants and mammals will require a more complete understanding of the factors that mediate cohesion. The rigorous experimental approaches we developed to elucidate the contributions of *C. elegans* meiotic kleisins can be applied to define the precise contributions of kleisin subunits in these other species.

## Materials and methods

### Strains

Worms strains were cultured using standard methods (Brenner, 1974). N2 Bristol was used as wild-type; other strains used in this study are listed in Supplementary file 1. Many mutants used in this study produce viable progeny with polyploid genomes; experiments using these alleles were performed on homozygous worms produced by known diploid, heterozygous parents.

### RNA Interference

The template for *air-2* dsRNA production was PCR amplified from the cDNA clone yk483g8 with T7 and T7_T3 primers (Supplementary file 2). The templates for *chk-1* and *chk-2* dsRNA production were amplified from the Open Biosystems RNAi library (Fisher Scientific, Pittsburgh, PA) clones GHR-10020 and GHR-11002, respectively. Other templates for dsRNA production were amplified from genomic DNA with gene-specific primers that included 5′ T7 sequences (Supplementary file 2). In all cases, PCR products were gel purified, then reamplified with T7 primers. dsRNA was prepared by in vitro transcription (Ambion, Austin, TX). Young adult hermaphrodites were injected with dsRNA at concentrations of 2.5–5 mg/ml, then mated with *him-8*; *mIs10* males at 20°C. Worms and embryos were fixed and stained 72 hr post injection for depletion of AIR-2, ATL-1, and SMC-1, 60 hr post injection for depletion of CHK-1 and CHK-2, and 48 hr post injection for depletion of SCC-1.

### Microscopy

Immunofluorescence analysis was performed as described previously (Chan et al., 2004). The following antibodies were used: rabbit anti-AIR-2 (Schumacher et al., 1998), rat anti-SMC-3 (Chan et al., 2003), rabbit anti-REC-8 and SCC-1 (Novus Biologicals, Littleton, CO), and mouse anti-REC-8 (CIM, Arizona State University). Anti-COH-3/4 antibodies were raised in rabbits (Covance, Princeton, NJ) immunized with a mixture of the peptides CGGNIDLLSTDDSEDIDDLAMADF and CGGNIDLLSTDDIEDIDDLAMADF (synthesized by D. King of the HHMI Mass Spectrometry Facility, University of California, Berkeley, CA). Peptides were coupled to Sulfolink (Thermo Fisher Scientific, Rockford, IL) for affinity purification. The staining pattern with this antibody was identical to that obtained with a commercial COH-3 antibody (Novus Biologicals, Littleton, CO) except that the nucleoplasmic background was much lower with our antibody.

LacI-His_6_-GFP (Darby and Hine, 2005) was expressed in BL21 *Escherichia coli* and purified on TALON resin (Clontech, Mountain View, CA). Animals heterozygous for the integrated *lacO* array *syIs44* were generated by crossing *syIs44* males to hermaphrodites that lacked the array. This crossing scheme allows self progeny of the hermaphrodite to be identified by the absence of GFP::LacI staining, ensuring that all examples of two GFP foci in the same nucleus resulted from defective SCC establishment in *syIs44* heterozygotes rather than a recombination defect in *syIs44* homozygotes. For quantification of distances between sister chromatids, Z-stacks of 1024 × 1024 pixel, unbinned images were acquired at 0.2 µm axial spacing on a Deltavision microscope (Applied Precision, Issaquah, WA) equipped with a 100x/1.4 NA objective lens. The X, Y, and Z positions of GFP foci were marked by hand in ImageJ and the distance between spots calculated by the three dimensional generalization of the Pythagorean theorem. Distances were measured in the −1 and −2 oocyte and in mid and late pachytene nuclei. For the analyses presented here, data were combined into ‘diakinesis’ and ‘pachytene’ datasets. In synapsis-defective mutants, distances were measured in nuclei that occupied similar positions in the gonad as mid and late pachytene nuclei in wild-type animals. To determine the effects of γ-irradiation on meiotic SCC, L4 hermaphrodites were exposed to 12 Gy from a sealed ^137^Cs source as described previously (Mets and Meyer, 2009), then fixed and stained 18 hr after exposure.

## Additional files

Supplementary file 1. Strains used in this study. **DOI:** http://dx.doi.org/10.7554/eLife.03467.020

Supplementary file 2. Oligos for amplification of dsRNA. **DOI:** http://dx.doi.org/10.7554/eLife.03467.021
