# Supplementary material for: Divergent kleisin subunits of cohesin specify mechanisms to tether and release meiotic chromosomes
Source: eLife. 2014 Aug 29;3:e03467. doi: 10.7554/eLife.03467 (PMC4174578; doi:10.7554/eLife.03467)
Supplement: Supplementary file 1. — Strains used in this study. DOI: http://dx.doi.org/10.7554/eLife.03467.020 [file elife03467s001.docx]

**Supplementary File 1. Strains used in this study**

| **Strain** | **Genotype** |
| --- | --- |
| CB0189 | *unc-32(e189) III* |
| TY3939 | *unc-32(e189) tim-1(t1545) III / hT2[qIs48] (I;III)* |
| TY5157 | *tim-1(t1545ts) III* |
| TY4986 | *htp-3(y428) ccIs4251[myo-3::GFP] I / hT2[qIs48] (I;III)* |
| TY4918 | *coh-3(gk112) V* |
| TY5115 | *coh-4(tm1857) V* |
| BS0913 | *unc-32(e189) glp-1(oz112) / unc-36(e251) glp-1(q175) III* |
| GZ0264 | *unc-119(ed3op) III; isIs17[pie-1p::pcn-1::gfp]* |
| TY5434 | *syIs44[pMH86(dpy-20(+)) + pPD49-78::lacI + lacO(256)] V* |
| TY4342 | *spo-11(me44) / nT1 IV; + / nT1[qIs51] V* |
| TY5167 | *spo-11(me44) / nT1 IV; syIs44 / nT1[qIs51] V* |
| TY5179 | *rec-8(ok978) / nT1 IV; + / nT1[qIs51] V* |
| TY5168 | *rec-8(ok978) / nT1 IV; syIs44 / nT1[qIs51] V* |
| TY4949 | *spo-11(me44) rec-8(ok978) / nT1 IV; + / nT1[qIs51] V* |
| TY5225 | *spo-11(me44) rec-8(ok978) / nT1 IV; syIs44 / nT1[qIs51] V* |
| TY5120 | *+ / nT1 IV; coh-4(tm1857) coh-3(gk112) / nT1[qIs51] V* |
| TY5433 | *+ / nT1 IV; coh-4(tm1857) syIs44 coh-3(gk112) / nT1[qIs51] V* |
| TY5425 | *spo-11(me44) / nT1 IV; coh-4(tm1857) coh-3(gk112) / nT1[qIs51] V* |
| TY5437 | *spo-11(me44) / nT1 IV; coh-4(tm1857) syIs44 coh-3(gk112) / nT1[qIs51] V* |
| TY5121 | *rec-8(ok978) / nT1 IV; coh-4(tm1857) coh-3(gk112) / nT1[qIs51] V* |
| TY5436 | *rec-8(ok978) / nT1 IV; coh-4(tm1857) syIs44 coh-3(gk112) / nT1[qIs51] V* |
| KR4941 | *atm-1(gk186) I* |
| TY5537 | *atm-1(gk186) I; syIs44 V* |
| TY5536 | *atm-1(gk186) I; rec-8 / nT1 IV; + / nT1[qIs51] V* |
| TY5535 | *atm-1(gk186) I; rec-8 / nT1 IV; syIs44 / nT1[qIs51] V* |
| TY5541 | *atm-1(gk186) I; + / nT1 IV; coh-4(tm1857) coh-3(gk112) / nT1[qIs51] V* |
| TY5539 | *atm-1(gk186) I; + / nT1 IV; coh-4(tm1857) syIs44 coh-3(gk112) / nT1[qIs51] V* |
| AV0146 | *chk-2(me64) rol-9(sc148) / unc-51(e369) rol-9(sc148) V* |
| TY5441 | *syIs44 chk-2(me64) rol-9(sc148)/syIs44 unc-51(e369) rol-9(sc148) V* |
| TY5432 | *rec-8(ok978) / nT1 IV; chk-2(me64) rol-9(sc148) / nT1[qIs51] unc-51(e369) rol-9(sc148) V* |
| TY5440 | *rec-8(ok978) / nT1 IV; syIs44 chk-2(me64) rol-9(sc148) / nT1[qIs51] unc-51(e369) rol-9(sc148) V* |
| TY5435 | *+ / nT1 IV; coh-4(tm1857) coh-3(gk112) chk-2(me64) rol-9(sc148) / nT1[qIs51] unc-51(e369) rol-9(sc148) V* |
| TY5439 | *+ / nT1 IV; coh-4(tm1857) syIs44 coh-3(gk112) chk-2(me64) rol-9(sc148) / nT1[qIs51] unc-51(e369) rol-9(sc148) V* |
| TY5456 | *+ / nT1 IV; syp-1(me17) / nT1[qIs51] V* |
| TY5455 | *+ / nT1 IV; syIs44 syp-1(me17) / nT1[qIs51] V* |
| TY5461 | *rec-8(ok978) / nT1 IV; syp-1(me17) / nT1[qIs51] V* |
| TY5460 | *rec-8(ok978) / nT1 IV; syIs44 syp-1(me17) / nT1[qIs51] V* |
| TY5458 | *spo-11(me44) rec-8(ok978) / nT1 IV; syp-1(me17) / nT1[qIs51] V* |
| TY5457 | *spo-11(me44) rec-8(ok978) / nT1 IV; syIs44 syp-1(me17) / nT1[qIs51] V* |
| VC0418 | *him-3(gk149) / nT1 IV; + / nT1[qIs51] V* |
| TY5464 | *him-3(gk149) / nT1 IV; syIs44 / nT1[qIs51] V* |
| AFS0002 | *him-3(gk149) rec-8(ok978) / nT1 IV; + / nT1[qIs51] V* |
| AFS0003 | *him-3(gk149) rec-8(ok978) / nT1 IV; syIs44 / nT1[qIs51] V* |
| TY5465 | *him-3(gk149) spo-11(me44) rec-8(ok978) / nT1 IV; + / nT1[qIs51] V* |
| TY5519 | *him-3(gk149) spo-11(me44) rec-8(ok978) / nT1 IV; syIs44 / nT1[qIs51] V* |
| TY5124 | *spo-11(me44) rec-8(ok978) / nT1 IV; coh-4(tm1857) coh-3(gk112) / nT1[qIs51] V* |
| TY5466 | *spo-11(me44) rec-8(ok978) / nT1 IV; coh-4(tm1857) syIs44 coh-3(gk112) / nT1[qIs51] V* |
