# Supplementary material for: Divergent kleisin subunits of cohesin specify mechanisms to tether and release meiotic chromosomes
Source: eLife. 2014 Aug 29;3:e03467. doi: 10.7554/eLife.03467 (PMC4174578; doi:10.7554/eLife.03467)
Supplement: Supplementary file 2. — Oligos for amplification of dsRNA. DOI: http://dx.doi.org/10.7554/eLife.03467.021 [file elife03467s002.docx]

**Supplementary File 2. Oligos for amplification of dsRNA**

| **Name** | **Gene** | **Sequence (T7 in bold)** |
| --- | --- | --- |
|  |  |  |
| AFS12 | T7 | **TAATACGACTCACTATAGG** |
| AFS13 | T7_T3 | **TAATACGACTCACTATAGG**AATTAACCCTCACTAAAG |
| AFS490 | *atl-1* | **TAATACGACTCACTATAGG**AATCGACGAATTCCAACCAC |
| AFS491 | *atl-1* | **TAATACGACTCACTATAGG**GTCTATCGCCGAGTCCAAAA |
| AFS191 | *him-1/smc-1* | GCG**TAATACGACTCACTATAGG**GAAGGCAGAGAACAACTCGACTCAG |
| AFS192 | *him-1/smc-1* | CGC**TAATACGACTCACTATAGG**GATCAGCAGAACCTCCGGACATA |
| RC136 | *scc-1* | **TAATACGACTCACTATAGG**GATCCATGGAACGGAAATTTCCCGTATAG |
| RC137 | *scc-1* | **TAATACGACTCACTATAGG**GCGGCCGCTCCAGCGAATCCACTCTGGATAGGG |
|  |  |  |
